# Adaptive Admixture of HLA Class I Allotypes Enhanced Genetically Determined Strength of Natural Killer Cells in East Asians

**DOI:** 10.1093/molbev/msab053

**Published:** 2021-02-22

**Authors:** Zhihui Deng, Jianxin Zhen, Genelle F Harrison, Guobin Zhang, Rui Chen, Ge Sun, Qiong Yu, Neda Nemat-Gorgani, Lisbeth A Guethlein, Liumei He, Mingzhong Tang, Xiaojiang Gao, Siqi Cai, William H Palmer, Jonathan A Shortt, Christopher R Gignoux, Mary Carrington, Hongyan Zou, Peter Parham, Wenxu Hong, Paul J Norman

**Affiliations:** 1 Immunogenetics Laboratory, Shenzhen Blood Center, Shenzhen, Guangdong, P. R. China; 2 Department of Transfusion Medicine, School of Laboratory Medicine and Biotechnology, Southern Medical University, Guangzhou, Guangdong, P. R. China; 3 Central Laboratory, Shenzhen Baoan Women’s and Children’s Hospital, Shenzhen, Guangdong, P. R. China; 4 Division of Biomedical Informatics and Personalized Medicine, University of Colorado, Anschutz Medical Campus, Aurora, CO, USA; 5 Department of Structural Biology, Stanford University School of Medicine, Stanford, CA, USA; 6 Clinical Laboratory, Wuzhou Red Cross Hospital, Wuzhou, Guangxi, P. R. China; 7 Inflammatory Cell Dynamics Section, Laboratory of Integrative Cancer Immunology, Center for Cancer Research, National Cancer Institute, NIH, Bethesda, MD, USA; 8 Basic Science Program, Frederick National Laboratory for Cancer Research (FNLCR), Frederick, MD21702, and Ragon Institute of MGH, Cambridge, MA, USA; 9 Shenzhen Institute of Transfusion Medicine, Shenzhen Blood Center, Shenzhen, Guangdong, P. R. China; 10 Department of Immunology and Microbiology, University of Colorado, Anschutz Medical Campus, Aurora, CO, USA

**Keywords:** HLA class I, KIR, admixture, adaptive introgression, natural killer cells, infectious disease, East Asia

## Abstract

Human natural killer (NK) cells are essential for controlling infection, cancer, and fetal development. NK cell functions are modulated by interactions between polymorphic inhibitory killer cell immunoglobulin-like receptors (KIR) and polymorphic HLA-A, -B, and -C ligands expressed on tissue cells. All *HLA-C* alleles encode a KIR ligand and contribute to reproduction and immunity. In contrast, only some *HLA-A* and *-B* alleles encode KIR ligands and they focus on immunity. By high-resolution analysis of *KIR* and *HLA-A*, *-B*, and *-C* genes, we show that the Chinese Southern Han (CHS) are significantly enriched for interactions between inhibitory KIR and HLA-A and -B. This enrichment has had substantial input through population admixture with neighboring populations, who contributed *HLA class I* haplotypes expressing the KIR ligands B*46:01 and B*58:01, which subsequently rose to high frequency by natural selection. Consequently, over 80% of Southern Han *HLA* haplotypes encode more than one KIR ligand. Complementing the high number of KIR ligands, the CHS *KIR* locus combines a high frequency of genes expressing potent inhibitory KIR, with a low frequency of those expressing activating KIR. The Southern Han centromeric *KIR* region encodes strong, conserved, inhibitory HLA-C-specific receptors, and the telomeric region provides a high number and diversity of inhibitory HLA-A and -B-specific receptors. In all these characteristics, the CHS represent other East Asians, whose NK cell repertoires are thus enhanced in quantity, diversity, and effector strength, likely augmenting resistance to endemic viral infections.

## Introduction

Human leukocyte antigen (HLA) class I molecules are critical components of immunity, whose extreme variation associates with resistance and susceptibility to infection, multiple immune-mediated diseases, and some cancers (Dendrou et al. 2018). *HLA class I* genes are located in the *major histocompatibility complex* (*MHC)* of chromosome 6 and encode proteins that bind peptide fragments derived from intracellular protein breakdown and transport them to the cell surface. In doing so, they can communicate to the adaptive immune system’s T cells whether a tissue cell is healthy or unhealthy due to infection or cancer. Subsets of HLA class I allotypes additionally contain an externally facing amino acid motif that binds killer cell immunoglobulin-like receptors (KIR), facilitating interaction with NK cells of innate immunity.

KIR are expressed on the surface of NK cells and regulate their functions through binding to HLA class I ligands on other cells (Cooper et al. 2009; Long et al. 2013). The functions of these interactions are crucial in immunity to aid recognition and elimination of infected or tumorous tissue and in reproduction to regulate placentation and fetal development (Parham and Moffett 2013). In accordance with these critical and independent roles in human health, KIR and their HLA class I ligands are subject to natural selection, mediating their exceptional diversity across individuals, populations, and species (Prugnolle et al. 2005; Parham and Moffett 2013). Indeed, *KIR* and *MHC* are some of the fastest evolving genomic loci in higher primates (Guethlein et al. 2015). Correlating with direct impact on both NK cell development and effector function (Vivier et al. 2011; Freud et al. 2017), numerous studies have implicated combinatorial diversity of *KIR* and *HLA class I* alleles with the course of specific infectious and autoimmune diseases as well as the success of transplantation (Holzemer et al. 2017; Boudreau and Hsu 2018). Importantly, the quantity as well as quality of these interactions can influence individual responses to infection (Pelak et al. 2011; Boelen et al. 2018). Thus, the polymorphism of *KIR* and *HLA class I* has profound impact on human health. Underexplored are the scale and characteristics of *KIR* and *HLA class I* combinatorial diversity worldwide and the processes that shape this diversity.

NK cells express overlapping subsets of KIR that are acquired stochastically during their development (Andersson et al. 2009). During this process, the interaction of inhibitory KIR with HLA class I KIR ligands broadens and strengthens subsequent effector functions of the NK cell repertoire (Hoglund and Brodin 2010; Saunders et al. 2015; Bjorkstrom et al. 2016). This education process matures some NK cells, allowing them to respond effectively to specific instances of infection or cancer, and enhances the NK cell repertoire compared with those that develop using other more conserved pairs of ligands and receptors. Activating KIR recognizes the same ligands as inhibitory KIR but are more sensitive to peptide repertoire changes caused by infection (Holzemer et al. 2017; Bastidas-Legarda and Khakoo 2019). In these roles, the inhibitory KIR dominate, and in pregnancy where HLA-A and -B have no function, HLA-C is dominant because all expressed HLA-C are KIR ligands (Parham and Moffett 2013). Four mutually exclusive sequence motifs define the four HLA class I epitopes that are KIR ligands: C1 is carried by subsets of HLA-C and HLA-B allotypes. C2 is carried by the other allotypes of HLA-C. Bw4 is carried by subsets of HLA-A and -B allotypes. The A3/11 motif is carried by a subset of HLA-A allotypes (HLA-A*03 and A*11). Thus, only some HLA-A and -B allotypes are KIR ligands, and their main role is likely to diversify the NK cell response to pathogens.

The *KIR* locus on chromosome 19q13.4 varies in gene content, containing up to eight genes encoding inhibitory KIR and five encoding activating KIR (Wilson et al. 2000). Four of the inhibitory KIR and four activating KIR have well-characterized HLA-A, -B, or -C ligands. Two broad groups of *KIR* haplotypes are present in every human population. *KIR A* haplotypes carry all four of the HLA-class I-specific inhibitory receptors and are associated with resistance to infectious diseases (Bashirova et al. 2006). *KIR B* haplotypes are more variable in their gene number, carrying two or more genes for inhibitory receptors as well as various activating receptor genes, and favor fetal development (Parham and Moffett 2013). A recombination hotspot separates the *KIR* locus into two segments, termed *centromeric* and *telomeric* (Wilson et al. 2000). Two inhibitory receptors specific for HLA-C are encoded in the centromeric region, and two HLA-A and -B-specific receptors are encoded in the telomeric region. Additional to gene content variation, polymorphism of both receptors and ligands can directly affect NK cell activity (Guethlein et al. 2015). Thus, by varying the number, density, specificity, strength, or signal transduction properties of the receptor-ligand interaction, genetic variation of *KIR* and *HLA class I* can predetermine functional differences in NK cell repertoires between individuals. This genetic diversity is substantial among populations, as demonstrated with high-resolution genotyping (Nemat-Gorgani et al. 2018). In such detailed analysis, Asian populations are underrepresented.

Comprising 20% of the human population, the Chinese Han are the largest ethnic group in the world (Abdulla et al. 2009). The Han have a complex population history and are presently structured with the Northern and Southern Han forming two main subgroups that are separated geographically by the Yangtze River (Wen et al. 2004). The Southern Han originated through large-scale population movements from the north during the last 2,000 years in parallel with admixture with resident and neighboring populations (Wen et al. 2004; Hellenthal et al. 2014). Importantly, for the current study, a major genetic distinction between the Northern and Southern Han occurs in the *MHC* and localizes to the region that spans *HLA-A*, *-B*, and *-C* (Xu et al. 2009; Chen et al. 2016). The most significant component of this difference is the A*33:03-B*58:01-C*03:02 HLA class I haplotype, which is common in the Southern Han and remains conserved across multiple unrelated individuals (Chen et al. 2016). Such strong linkage disequilibrium is consistent with recent acquisition of this haplotype by admixture (Chen et al. 2016). This haplotype encodes two KIR ligands: HLA-B*58:01 and C*03:02 (Saunders et al. 2015). Although less is known of *KIR* allele diversity in the Han, several studies established that the genes characteristic of *KIR A* haplotypes are common and demonstrated differences in their distribution among the different Han groups and among other resident populations (Yao et al. 2011, 2019; Wang et al. 2012; Bao et al. 2013). These studies also confirmed that *KIR* and *HLA class I* combinatorial diversity is an important factor in pregnancy syndromes, infectious disease, blood cancers, and transplantation outcome in the Han. They also uncovered both similarities and differences from the specific disease associations observed in Europeans (Jiang et al. 2013; Long et al. 2015; Bao et al. 2016; Shen et al. 2016; Su et al. 2018). To investigate these findings, we have examined how demographic and evolutionary processes have shaped combinatorial diversity of HLA class I and KIR in the Chinese Southern Han (CHS).

## Materials and Methods

### Study Samples

Peripheral blood samples were collected from 306 unrelated healthy volunteer blood donors from Shenzhen, Guangdong, China. All donors self-identified to be of Han ethnicity from southern China. All subjects provided written informed consent for participation in the present research, which was approved by the ethics review board of Shenzhen Blood Center, Shenzhen, Guangdong, China.

### Genomic DNA Extraction

Genomic DNA was extracted from 400 μl of peripheral blood using a MegCore Nucleic Acid Extractor (MegCore, Taiwan, China). DNA purity and concentration were tested using a Nanodrop 2000 spectrophotometer (Thermo Scientific, Wilmington, Delaware USA) and adjusted to a concentration of 50–100 ng/μl.

### High-Resolution HLA-A, -B, and -C Genotyping

*HLA-A*, *-B*, and *-C* genotyping was performed using the AlleleSEQR HLA sequencing-based genotyping commercial kit (Atria Genetics, San Francisco, USA). According to the manufacturer’s instructions, exons 2–4 for *HLA-A*, *-B*, and *-C* were sequenced in both directions using an ABI 3730XL DNA sequencer (Applied Biosystems, Foster City, CA, USA). HLA genotypes were assigned using the Assign 4.7 software (Conexio Genomics, Fremantle, Australia). Samples giving ambiguous allele combinations by sequencing were further resolved using HLA PCR-SSP (Olerup, Stockholm, Sweden).

### High-Resolution KIR Genotyping

The presence or absence of *KIR2DL1*, *2DL2*/*3*, *2DL4*, *2DL5*, *2DS1*, *2DS2*, *2DS3*, *2DS4*, *2DS5*, *3DL1*/*S1*, *3DL2*, and *3DL3* was first determined for each individual using the “KIR Ready Gene” PCR-SSP kit (Inno-Train Diagnostik GmbH, Frankfurt, Germany). The *KIR* genes identified using PCR-SSP were then subject to nucleotide sequencing of all exons (Deng et al. 2019b). Sequencing reactions were performed using ABI PRISM BigDye Terminator Cycle Sequencing Ready reagents and analyzed using an ABI 3730 DNA Sequencer (Applied Biosystems, Foster City, USA). *KIR* alleles were assigned using Assign 4.7 allele identification software (Conexio Genomics, Fremantle, Australia) and release 2.6.1 (February 2015) of the Immuno-Polymorphism database (IPD) (Robinson et al. 2015). When the sequencing results gave ambiguous allele combinations, we used group-specific PCR primer pairs to amplify and sequence the target alleles separately (Zhang and Deng 2016).

### Novel KIR Alleles

To confirm and fully characterize any novel allele identified during amplicon sequencing, we cloned and sequenced *KIR* transcripts. Further peripheral blood samples were collected, and total RNA isolated using the Maxwell 16 low elution volume simplyRNA Blood Kit (Promega, Madison, USA). Complementary DNA (cDNA) was synthesized using the Transcriptor First Strand cDNA Synthesis Kit (Roche, Basel, Switzerland). *KIR* transcripts were amplified specifically from cDNA using primer pairs described previously (Yawata et al. 2006), with addition of KIR3DL3-specific primers (forward 5′-GGTTCTTCTTGCTGGAGGGGC-3′ and reverse 5′-TTACACGCTGGTATCTGTTGGGG-3′). The amplified transcripts were cloned using the TA cloning kit (Takara, Dalian, China), and at least three clones of any novel allele were sequenced. The sequences of novel *KIR* alleles were submitted to GenBank and the IPD KIR database (Robinson et al. 2015) to obtain official names.

### Haplotype and Ligand Frequencies

*KIR* and *HLA-A*, *-B*, and *-C* allele frequencies were calculated from the observed genotypes. The subsequent genotype distributions for all loci were consistent with Hardy–Weinberg equilibrium. *HLA class I (A–B–C)* haplotype frequencies were determined using the EM algorithm of Arlequin software version 3.5 (Excoffier and Lischer 2010). *KIR* haplotype frequencies were determined using PHASE II (Stephens and Donnelly 2003). The following parameters were used; -f1, -x5, and -d1, and from the output, the two haplotypes with highest probability were taken for each individual. For comparing *HLA class I* and *KIR* haplotypes with representative global populations, we used populations for which genotype data was available from every individual sampled, and for which the resolution of genotyping was the same as the CHS described here. Thus, we used data from the Ga-Adangbe from Ghana in West Africa (Norman et al. 2013), the Nama, a KhoeSan population from Southern Africa (Nemat-Gorgani et al. 2018), Yucpa from South America (Gendzekhadze et al. 2009), Europeans from the USA (Norman et al. 2016), Māori from Oceania (Nemat-Gorgani et al. 2014), and Hondo Japanese (Yawata et al. 2006). Specifically, for the *HLA class I* analyses, these data sets were supplemented with a subset of populations described in the 13th International Histocompatibility Workshop and Conference report (Meyer et al. 2007). Inclusion criteria for the latter were East/Southeast Asian populations having >90 *HLA-A*, *-B*, and *-C* genotyped individuals and one East African group that we chose at random from three present (Nandi from Kenya). We compared the proportion of *HLA class I* haplotypes encoding one KIR ligand to those encoding more than one KIR ligand across populations using a two-proportion *Z* test, using the prop.test function in R (R Development Core Team 2008). Watterson’s homozygosity *F* test was performed using Pypop software (Lancaster et al. 2007), with 10,000 replicates to calculate the normalized deviate *F*_nd_ test (Salamon et al. 1999).

### Comparison of HLA Class I and KIR Ligand Distributions

We obtained genome-wide SNP data from the 1,000 Genomes Project Phase 3 individuals (Auton et al. 2015) from whom *HLA* allele calls are available (Abi-Rached et al. 2018). To confirm that chromosome 6 SNPs correlate with genome-wide SNPs, PCAs were constructed using the PCA function in PLINK (Purcell et al. 2007). Genotype data was first filtered to include only SNPs having minor allele frequency >1% and that were independent of other SNPs (linkage disequilibrium, *r*^2^ < 0.3). The correlation between PC1 calculated from whole genome and chromosome 6 genotypes is 0.996. We then calculated the respective Euclidean distances between populations from the frequencies of chromosome 6 SNPs, *HLA class I* alleles, and the number of KIR ligands encoded per haplotype. To compare the relationship between global distribution of human genetic variation and *HLA* allele frequencies to the relationship between global distribution of human genetic variation and the number of KIR ligands per haplotype, we determined the correlation of each respective topology with the topology for the full set of chromosome 6 SNPs, using the *dendextend* package (v1.14.0) (Galili 2015).

### Admixture Estimates

Whole genome SNP genotypes for Japanese (*N* = 104), Vietnamese (*N* = 99), Han from Beijing (*N* = 103), Southern Han (*N* = 105), and Dai (*N* = 93) were obtained from the 1,000 Genomes (Phase 3) Project (Auton et al. 2015). We used any SNPs having minor allele frequency >1% and independent of other SNPs (linkage disequilibrium, *r*^2^ < 0.3). Admixture was calculated for chromosome 6 using the ADMIXTURE program (Alexander et al. 2009), with the unsupervised option and *k* = 3. Two regions were analyzed, the *MHC* (chr6:28,477,797–33,448,354: 3,541 SNPs) and chromosome 6 excluding the *MHC* (84,898 SNPs). The number of SNPs required to resolve genetic ancestry is inversely proportional to the genetic distance between populations (Alexander et al. 2009). To ensure that there was adequate population structure for admixture estimates, we therefore calculated the *F*_ST_ for chromosome 6 across the populations used to calculate genetic ancestry. We first filtered out variants with a minor allele frequency less than 1% and then calculated *F*_ST_ for each SNP variant of chromosome 6 using VCF tools 0.1.12b, which implements a modified version of Wright’s *F*_ST_ (Danecek et al. 2011). Mean *F*_ST_ was then determined for the variants of chromosome 6, within and outside the *MHC.* We selected a *K* of 3 to represent the three primary ancestry groups in the region that are represented in the 1,000 Genomes data: Japanese, South East Asian, and East Asian (Chen et al. 2016). *HLA class I* alleles were obtained from the 1,000 Genomes Project data (Gourraud et al. 2014). We analyzed the Hondo Japanese (JPT), Vietnamese (KHV), Chinese Dai (CDX), CHS, and Beijing Han (CHB). Validating their use for this purpose, the correlation of the *HLA class I* allele frequencies between our study population and the CHS is 0.95 (*P* = 6.65^−11^, supplementary material S1, Supplementary Material online). Individuals were considered carriers if they had at least one copy of the respective allele. Distributions of ancestry proportions for carriers and noncarriers of specific *HLA* alleles were compared using a Wilcoxon test, using the wilcox.test function in R (R Development Core Team 2008).

### Estimates of Nucleotide Diversity

We used π (Nei and Takahata 1993) to measure the nucleotide diversity of haplotypes carrying specific *HLA-B* alleles. We used the phased genomes of the CHS population available from the 1,000 Genomes Project (Auton et al. 2015) and extracted the genomic region containing the *HLA-B* and *-C* genes, with 500 kbp flanking on each side. For each carrier of a given allele, we identified (by sequence) and retained the haplotype representing the allele of interest. For each given allele, we pooled all of the respective haplotypes present in the population and calculated π in 100 bp windows using VCFtools (Danecek et al. 2011). Distributions of π values were compared between respective alleles with a Wilcoxon test using the wilcox.test function in R.

### Tests for Positive Selection Affecting Specific HLA Class I Alleles

We filtered 1,000 Genomes genotyping data of chromosome 6 from the CHS population to remove nonbiallelic and duplicated SNPs (Purcell et al. 2007) and then phased using the program Eagle (Loh et al. 2016). We used the program Selscan (Voight et al. 2006) to calculate the integrated haplotype statistic (iHS). The statistic is a measure of haplotype diversity associated with a given genetic variant, where lower diversity and longer haplotypes correlate with selection of that variant. Selscan reports iHS that are either positive or negative based on whether the variant exhibiting the extended haplotype homozygosity is ancestral or derived, respectively. The characterization of ancestral or derived can be confounded by population size or by long-term balancing selection (as observed for HLA). For this reason and because we are specifically interested in the distribution of iHS for variants on specific *HLA class I* haplotypes, regardless of whether selection is targeting an ancestral or derived variant, we used the absolute value of iHS.

To determine if specific *HLA class I* alleles have been targeted by directional selection in the CHS, we again used the 1,000 Genomes SNP data from the CHS population. SNPs within the following hg19 coordinates were used: *HLA-A*, Chr6: 29,910,089–29,913,770; *HLA-B*, Chr6: 31,321,648–31,325,007; *HLA-C*, and Chr6: 31,236,517–31,239,917. We phased haplotypes from individuals positive for each given *HLA class I* allele and aligned them to reference sequences to identify the haplotype containing that allele. The alignments were then used to identify “tagging” SNPs that could be used to identify each given *HLA class I* allele. The criteria for choosing tagging SNP alleles were that they must be present in every individual carrying the corresponding *HLA class I* allele and that they must be absent from the other *HLA class I* alleles in the analysis. We analyzed the alleles present on the 10 most frequent *HLA class I* haplotypes that we observed in the CHS; HLA-B*15:02 was excluded because we were not able to identify unique tagging alleles on haplotypes carrying this allele. For each tagging SNP, we calculated the integrated haplotype score (iHS) using SelScan. We used the absolute value of iHS since derived alleles under selection will have a negative value and ancestral alleles under selection will have a positive value (Szpiech and Hernandez 2014). Using a Wilcoxon two-sample test, we examined whether the distributions of absolute iHS values differed between tagging SNPs of *HLA* alleles and SNPs of the full chromosome 6.

### Assessment of Receptor/Ligand Quality and Quantity

As described previously (Nemat-Gorgani et al. 2018), experimental data were used to determine the interacting pairs of KIR and HLA class I, which are listed in supplementary material S2 (Supplementary Material online). To determine the quantity of receptor/ligand interactions, the number of KIR/HLA allotype pairs that are known to interact was summed for each individual, and homozygous KIR or HLA allotypes were counted twice. To determine the diversity of interactions, the number of different KIR/HLA allotype pairs that are known to interact were summed for each individual (in this case, homozygous allotypes were counted once). Populations were compared using unpaired *t* tests, using GraphPad software.

## Results

### High Frequency of KIR Ligands in the Southern Han

**Fig. 1.**
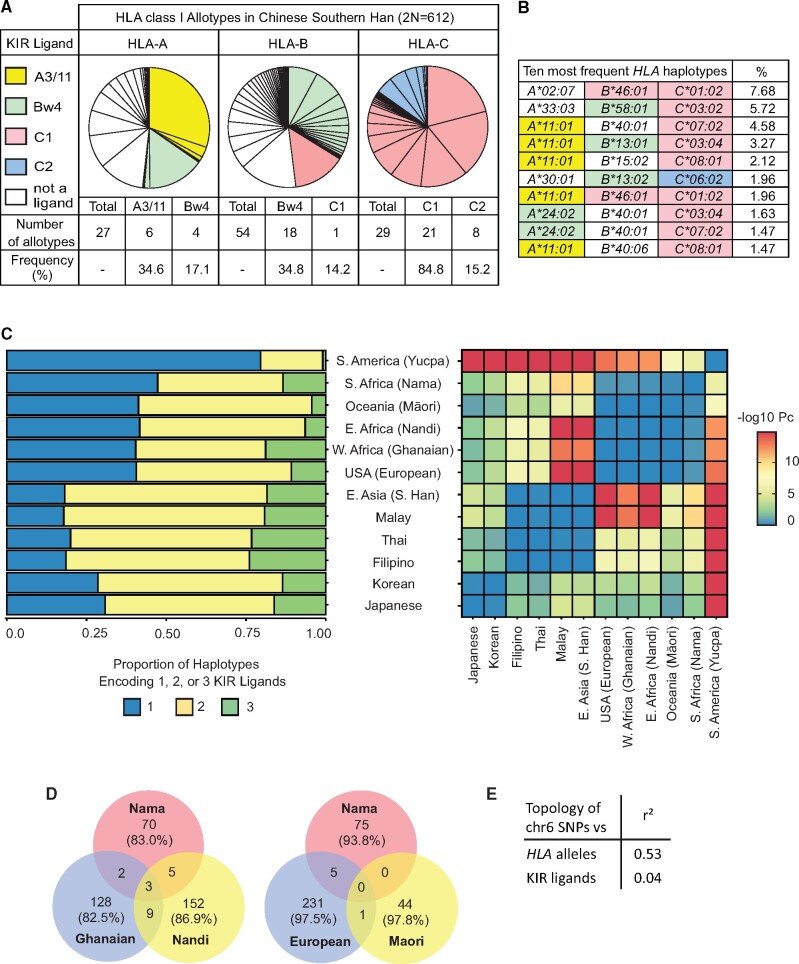
Chinese Southern Han *HLA class I* haplotypes express multiple KIR ligands. (*A*) Pie charts show the frequency spectra for HLA-A, -B and -C allotypes of the Southern Han cohort of 306 unrelated individuals (2N = 612). Each pie segment represents one allotype. Alternative sequence motifs in the α1 domain of the HLA class I molecule determine the four epitopes recognized by different KIR, and which are also called KIR ligands. The A3/11 epitope is carried by HLA-A3 and -A11 (yellow-colored pie segments); the Bw4 epitope is carried by subsets of HLA-A and -B allotypes (green-colored pie segments). The C1 epitope is carried by a majority of HLA-C allotypes as well as by HLA-B*46 and HLA-B*73 (red-colored pie segments). The C2 epitope is carried by all HLA-C allotypes that do not carry C1 (blue-colored pie segments). Clear pie segments correspond to allotypes that are not KIR ligands. Supplementary material S2 (Supplementary Material online) lists all the HLA-A, -B and -C allotypes present in the study population and shows which KIR ligand motifs they carry. (*B*) Shows the 10 most frequent *HLA class I* haplotypes in the Southern Han and their frequencies (2N = 612). Colored shading indicates *HLA class I* alleles that encode KIR ligands, as described in panel A. (*C*) (left) Bars show the combined frequencies of *HLA class I* haplotypes encoding one (blue), two (gold) or three (green) KIR ligands in seven representative populations worldwide (Southern, Western and Eastern Africa, Europe, Oceania, South America and East Asia) and five further East/South East Asian populations. (Right) Heat-plot shows pairwise comparisons between populations of the proportion of *HLA class I* haplotypes encoding one KIR ligand to those carrying two or more KIR ligands. As shown in the key, colors correspond to −log^10^ of a Benjamini–Hochberg corrected p (Pc) from pairwise comparisons. (*D*) Venn diagrams show the distribution of *HLA class I* haplotypes within representative subsets of populations. The number of haplotypes in each overlapping region is given. The % values indicate the combined frequency of haplotypes unique to a population when compared with the other populations in the diagram. (*E*) Shows correlation (*r*^2^) of the topology obtained from the 1000 Genomes populations according to the SNP frequencies of chromosome 6 compared with that obtained from HLA allele frequencies and KIR ligand frequencies. Correlations of topologies are shown and dendrograms given in supplementary material S5 (Supplementary Material online).

All HLA-C and subtypes of HLA-A and -B allotypes are ligands for KIR, which are expressed on the surface of NK cells to modulate their functions in immunity and reproduction. To characterize the distribution of KIR ligands in the CHS, we analyzed the *HLA-class I* genes of 306 healthy individuals. We identified 27 *HLA-A*, 54 *HLA-B*, and 29 *HLA-C* alleles (supplementary material S3, Supplementary Material online). Each of these 110 alleles encodes a different HLA class I allotype and 58 of them are known KIR ligands (fig. 1*A*). The majority of 233 *HLA class I* haplotypes, including the ten most frequent (fig. 1*B*), encode more than one KIR ligand (70.3% of distinct haplotypes; 81.8% by frequency, supplementary material S4*A*, Supplementary Material online). To investigate the unusually high frequency of KIR ligands, we compared Southern Han *HLA class I* haplotypes with those of sub-Saharan African, Oceanian, European, and South American populations that represent major modern human groups (Rosenberg et al. 2002; Tishkoff et al. 2009). Among the seven populations, 831 different *HLA class I* haplotypes were observed (supplementary material S4*B*, Supplementary Material online). Five of the major populations have a similar distribution of KIR ligands, with each population having an approximately equal frequency of *HLA class I* haplotypes carrying one and two KIR ligands, and a smaller frequency of haplotypes carrying three KIR ligands (fig. 1*C*). Only Southern Han and South Americans differ from this pattern, with the Han encoding more and the Amerindians encoding less KIR ligands per haplotype than other populations (fig. 1*C*). The difference in the proportion of *HLA class I* haplotypes encoding one versus more than one KIR ligand between the Southern Han and each of the other representative populations is statistically significant, as is that between Amerindians and the other populations (two-proportions *Z*-test, Benjamini–Hochberg corrected *P* < 0.001, fig. 1*C*). The allele frequency distribution of South American Amerindians was likely influenced by severe population bottlenecks, leading to a reduced genome-wide diversity compared with other populations (Fagundes et al. 2008; Raghavan et al. 2015), whereas the Han were not subject to severe population-specific bottleneck (Henn et al. 2012; Schiffels and Durbin 2014; Lu et al. 2016). Finally, to examine if the CHS are representative of other related populations, we examined HLA data obtained from East Asian (Hondo Japanese and Korean) and Southeast Asian (Thai, Malay, and Filipino) populations (Meyer et al. 2007). This analysis showed that these populations also have a high frequency of *HLA class I* haplotypes encoding multiple KIR ligands (fig. 1*C*). Our analysis thus shows that East Asian and South East Asian *HLA class I* haplotypes encode more ligands for inhibitory KIR than the haplotypes of any other populations.

Despite having distinct population histories, the three sub-Saharan African, Oceanic, and European populations all have a similar mean number of KIR ligands per *HLA* haplotype (fig. 1*C*). However, very few *HLA class I* haplotypes are shared by any of these populations. For example, only 19 of 369 haplotypes detected in Africans are present in more than one of the three African populations studied, and comparing the disparate Southern African Nama, Māori, and European populations revealed just six haplotypes in common (fig. 1*D*). We therefore examined whether the observed distribution of *HLA class I* encoded KIR ligands is consistent with the global distribution of human genetic variation. We analyzed genome-wide SNP and *HLA* allele data of individuals from the 1000 Genomes Project (Auton et al. 2015; Abi-Rached et al. 2018). Trees were generated based on the frequencies of all chromosome 6 SNPs, *HLA class I* alleles, and the number of KIR ligands encoded by *HLA* haplotypes (supplementary material S5, Supplementary Material online). We observed a modest correlation (*r*^2^ = 0.53) between the topology obtained from chromosome 6 SNPs and that obtained from *HLA* allele frequencies (fig. 1*E*), consistent with combined effects of local adaptation and human demography in shaping global HLA diversity (Prugnolle et al. 2005; Solberg et al. 2008). By contrast, we observed little correlation (*r*^2^ = 0.04) between chromosome 6 SNPs and the number of KIR ligands per haplotype. Thus, whereas genetic patterns arising from human demography are broadly congruous with observed HLA diversity, they are a poor predictor of KIR ligand composition.

### The CHS Acquired HLA Haplotypes Encoding Multiple KIR Ligands by Admixture

**Fig. 2.**
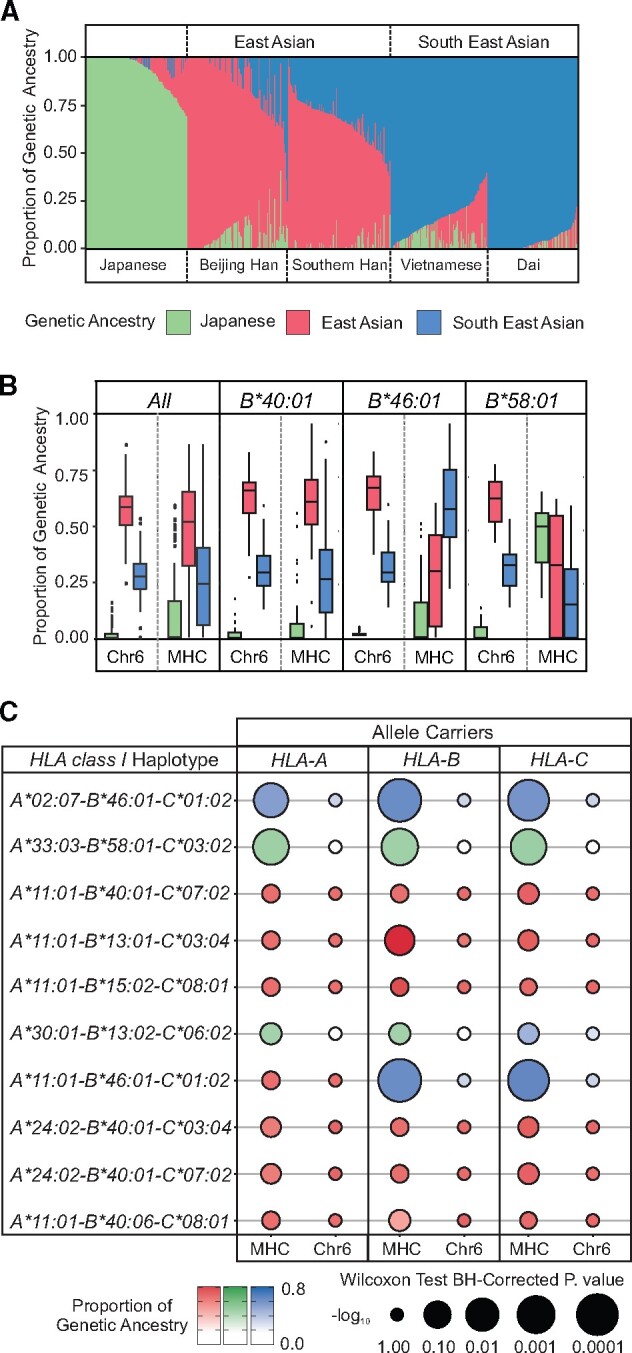
HLA-B*46:01 and -B*58:01 were acquired by admixture into the Chinese Southern Han. (*A*) Shown are the relative proportions of genetic ancestry among Asian populations from the 1000 genomes project, plotted by considering three ancestral groups [K = 3: Japanese (green), East Asian (red) and South East Asian (blue)]. (*B*) Shown are the relative proportions for each of the three genetic ancestries in chromosome 6 excluding the *MHC* (left) and within the *MHC* (right) for selected Chinese Southern Han individuals, shown from left to right; all individuals, B*40:01 carriers, B*46:01 carriers, and B*58:01 carriers. (*C*) Shown for each of the ten most frequent *HLA class I* haplotypes in the Chinese Southern Han is a comparison of mean admixture proportion of the genetic ancestry group that is most abundant in *MHC* compared with the proportion of that ancestry in chromosome 6 with *MHC* excluded. The size of the circle represents the −log_10_ (Benjamini–Hochberg corrected p) from a Wilcoxon test. The difference in size and shade between the two circles corresponds to extent and direction, respectively, of any shift in the genetic ancestry proportions between the *MHC* and the remainder of the chromosome 6.

Previous analyses suggested that specific *MHC* genomic region haplotypes (which include the *HLA* genes) present in the CHS were obtained from the Northern Han through admixture (Chen et al. 2016). The most frequent HLA class I allotypes contributing to the enrichment of KIR ligands in the CHS are HLA-A*11, -A*24, -B*46, and -B*58 (fig. 1*B*). We therefore examined the relative contributions of admixture to the high frequency of these alleles in the CHS. For this analysis, we considered known admixture events (Wen et al. 2004; Xu et al. 2009; Hellenthal et al. 2014) and drew upon the 1,000 Genomes SNP and *HLA* genotype data (Gourraud et al. 2014; Auton et al. 2015) from Hondo Japanese, Vietnamese, Dai, Beijing Han, and CHS. Consistent with the previous work examining whole-genome data (Takeuchi et al. 2017), in analyzing chromosome 6, we identified three primary genetic ancestries, corresponding to the Japanese, East Asian (Southern and Beijing Han), and South East Asian (Vietnamese and Dai) population groups (fig. 2*A*). That we identify a higher “Japanese” component in the Beijing than Southern Han (36% vs 22%: fig. 2A) likely reflects the higher proportion in Beijing of Northern Han (Auton et al. 2015), a population from which we have no data for the current study. Supporting this observation, the greatest contribution from China to Japanese ancestry is from the Northern Han (Chen et al. 2016; Takeuchi et al. 2017).

We compared the relative proportions of the three genetic ancestries in the CHS within the *MHC* region of chromosome 6 to their proportions throughout chromosome 6 excluding the *MHC*. This analysis revealed a predominance of East Asian ancestry throughout the length of chromosome 6, including the *MHC* (fig. 2*B*). In carriers of HLA-B*40:01, the most frequent *HLA-B* allele in CHS, there is also clear East Asian ancestry throughout the length of chromosome 6 (fig. 2*B*). The proportion of East Asian ancestry is similar in B*40:01 carriers than noncarriers (Wilcoxon test, *P* = 0.98). By contrast, among HLA-B*46:01 carriers, the *MHC* is primarily of South East Asian ancestry (fig. 2*B*) with carriers having a significantly higher proportion of South East Asian genetic ancestry in the *MHC* than outside the *MHC* (*P* = 2.7^−6^), or within the *MHC* of noncarriers (*P* = 1.9^−5^). Similarly, among HLA-B*58:01 carriers, the *MHC* region is primarily of Japanese ancestry (fig. 2*B*), with carriers having a significantly higher proportion of Japanese genetic ancestry within the *MHC* than outside the *MHC* (*P* = 2.4^−4^) and compared with non-B*58:01 carriers (*P* = 6.1^−7^). For each carrier group, the primary genetic ancestry outside of the *MHC* region was determined as East Asian (fig. 2*B*). We calculated the mean *F*_ST_ across the East Asian, South East Asian and Japanese ancestral groups, both within and outside the *MHC*. Further supporting the observed population structure as specific to the *MHC* region, among the three ancestral groups the *F*_ST_ values range from 0.098 to 0.161 within the *MHC*, compared with 0.012–0.017 for the remainder of chromosome 6.

Based on the analysis of HLA-B*46 and -B*58, we examined the genetic ancestry proportions of carriers of any alleles that comprise the ten most frequent *HLA class I* haplotypes observed in the CHS. For every group of individuals defined by the allele they carried, the primary genetic ancestry outside of the *MHC* was determined as East Asian. We then determined the primary genetic ancestry in the *MHC* region for carriers of each respective allele and then determined the relative proportion of that ancestry in the remainder of the *MHC*. This analysis identified six haplotypes maintaining strong evidence of East Asian genetic ancestry both within the *MHC* and throughout chromosome 6. These haplotypes include those that carry A*11:01 and A*24:02 as well as B*15:02 and B*40:01 (fig. 2*C*). It was shown previously that these alleles all likely derive from introgression with archaic humans (Abi-Rached et al. 2011), and our results and others (Solberg et al. 2008; Gonzalez-Galarza et al. 2015) suggest that these haplotypes are now endemic to East Asia. The analysis also identified four haplotypes having genetic ancestry within the *MHC* that is distinct from the ancestry of the remainder of the chromosome (fig. 2*C*). For three of the haplotypes, which include the two most frequent haplotypes in the population, this distinction is statistically significant (*P*_corr_ < 0.01). Two of these haplotypes contain B*46:01 and one contains B*58:01 (fig. 2*C*). In total, four of five of the *HLA-B* alleles that encode a KIR ligand and are present on these ten most frequent haplotypes show increased evidence for admixture in the *MHC* region. By contrast, neither of the two *HLA-A* alleles that encode a KIR ligand show a genetic ancestry within the *MHC* that differed from the East Asian ancestry throughout chromosome 6. This finding suggests that the number of *HLA-B* alleles encoding KIR ligands was enhanced in CHS by admixture with neighboring or displaced populations. In summary, these findings clearly show that the B*46:01 and B*58:01 alleles are present in CHS through admixture.

### Admixed HLA Haplotypes Have Increased in Frequency under Positive Natural Selection in the CHS

**Fig. 3.**
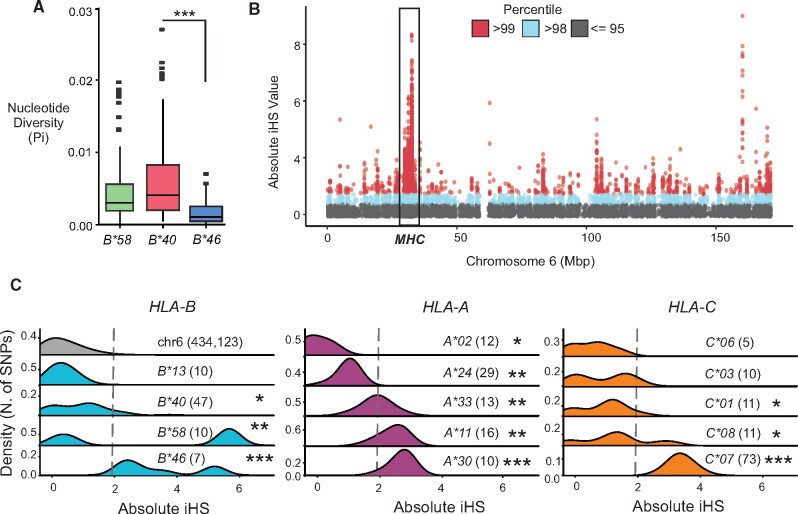
Positive selection has targeted *HLA class I* genes in the Chinese Southern Han. (*A*) Shown is the nucleotide diversity (π) of genomic sequence ±500 kbp of the *HLA-B* and *-C* genes for haplotypes containing the specific *HLA-B* alleles, B*40, B*46, and B*58. π was measured in windows of 100 bp. ***p < 0.001 by Wilcoxon test. (*B*) Manhattan plot shows the absolute iHS values above the 95th percentile calculated for independent SNPs throughout chromosome 6 in Chinese Southern Han. The *MHC* region is boxed. (*C*) Density plots show the distribution of absolute iHS values for chromosome 6 (top left, grey shading), and for SNPs unique to haplotypes carrying specific *HLA-B* (left, cyan), *HLA-A* (center, purple) and *HLA-C* (right, orange) alleles, as indicated in each plot. The number of SNPs unique to each of the *HLA* alleles is shown in brackets next to the allele name. For each allele, the distribution of iHS values was compared with the distribution on chromosome 6 using a Wilcoxon two-sample test (*P < 0.05, **P < 0.05^−5^, ***P < 0.05^−10^). Grey dashed line marks the 95th percentile of absolute iHS values for chromosome 6 SNPs (=1.93). The density shown is the kernel density estimate of the SNP counts associated with the distribution of absolute iHS values.

To investigate whether or not the admixed haplotypes were also subject to natural selection, we examined further characteristics of their diversity and distribution. We first measured nucleotide diversity (π) of the genomic sequence flanking ±500 kb of specific *HLA-B* alleles (fig. 3*A*). We found significantly reduced nucleotide diversity of haplotypes containing HLA-B*46 compared with haplotypes containing HLA-B*40 (mean π of B*40 =* *2.2 × 10^−3^, B*46 =* *0.6 × 10^−3^, Wilcoxon test, *P* = 1.24 × 10^−12^). We also observed that haplotypes containing B*58 have lower diversity than B*40, but this reduction was not statistically significant (mean π of B*58 =* *1.6 × 10^−3^, Wilcoxon test, *P* = 0.12). This reduced diversity suggests that admixed B*46 haplotypes have arisen in frequency in the CHS without accumulating mutations. To further explore this finding, we used the iHS statistic, which identifies genomic variants that have increased in frequency recently and rapidly under natural selection, so that their haplotypic background has not yet been diversified by recombination (Voight et al. 2006). We identified a strong signal of recent selection (iHS ≥ 99th percentile) that falls precisely in the *MHC* of the CHS (fig. 3*B*).

To investigate the patterns of selection specific to *HLA class I* alleles that are common in the CHS, we first identified SNPs that characterize the alleles present on the ten most frequent haplotypes and then compared their distributions of iHS values to the distribution of all the SNPs of chromosome 6. In this comparison, the B*13, C*03, and C*06 alleles did not exhibit iHS values higher in magnitude than the mean for chromosome 6 (fig. 3*C*), and we did not find any SNPs unique to B*15. By contrast, for B*46, the mean absolute iHS of 3.38 was significantly higher than the mean for chromosome 6 of 0.785 (Wilcoxon two-sample test, *P* = 5.77^−6^), as was the mean iHS for B*58 (3.43, *P* = 1.8^−3^). Although the signal for B*58 is weaker, there is a more distinct subset of SNPs having extremely high iHS values (>99th percentile, fig. 3*C*), which could indicate recent selection of an older haplotype, although it was not possible from our analysis to determine if the SNP allele was ancestral or derived in each case. This analysis identified two other *HLA class I* alleles as having highly distinct signatures of directional selection, A*30 and C*07. HLA-C*07 is known to interact strongly with KIR to educate NK cells (Yawata et al. 2006; Hilton et al. 2015a). Although HLA-A*30 does not encode a KIR ligand, it occurs on the same haplotype as HLA-B*13 (fig. 1*B*), which is not selected by this measure (fig. 3*C*) but does carry a KIR ligand. By contrast, although we were unable to identify adequate tag SNPs for B*15 alleles, the HLA-A*11 and -C*08 alleles that encode KIR ligands and comprise the haplotype carrying B*15:02 do show evidence of natural selection (fig. 3*C*).

Interestingly, the mean iHS for B*40 associated SNPs was also significantly higher than the chromosome average (1.88, *P* = 7.7^−6^). However, fewer B*40-specific SNPs had an iHS value in the 95th percentile than B*58 or B*46-specific SNPs (30, 50, and 100% of SNPs, respectively). Importantly, the most frequent B*40 containing haplotypes in the CHS carry either A*11 or A*24, which are KIR ligands (fig. 1*B*). Again, this analysis showed both A*11:01 (mean = 2.8, Wilcoxon two sample test *P* = 1.45^−11^) and A*24:02 (mean = 1.37, *P* = 1.27^−9^) associated SNPs have significantly higher iHS values than the chromosome average, with A*11 having a mean iHS that is above the 95th percentile. Haplotypes carrying HLA-A*11:01, A*24:02, B*46:01, or B*58:01 were previously identified to have unusually high LD in this population (Chen et al. 2016). Together, these findings illustrate that multiple HLA class I allotypes present in the CHS have been targeted by natural selection and suggest that one major consequence has been an increase in the number of KIR ligands present in the population.

In summary, these results show that similar quantities of KIR ligands can be obtained across populations using different subsets of *HLA class I* haplotypes, indicating that there is pressure to maintain a certain ratio of KIR ligands, regardless of the background HLA allotype, and that this ratio is altered in East Asia. Our observations show that successive rounds of admixture followed by natural selection favoring specific *HLA class I* haplotypes have led to an increased quantity of KIR/HLA interactions in East Asian populations. To investigate the characteristics of this receptor and ligand diversity, we next studied the *KIR* locus in the CHS.

### High Frequency of Inhibitory KIR Allotypes in Southern Han

**Fig. 4.**
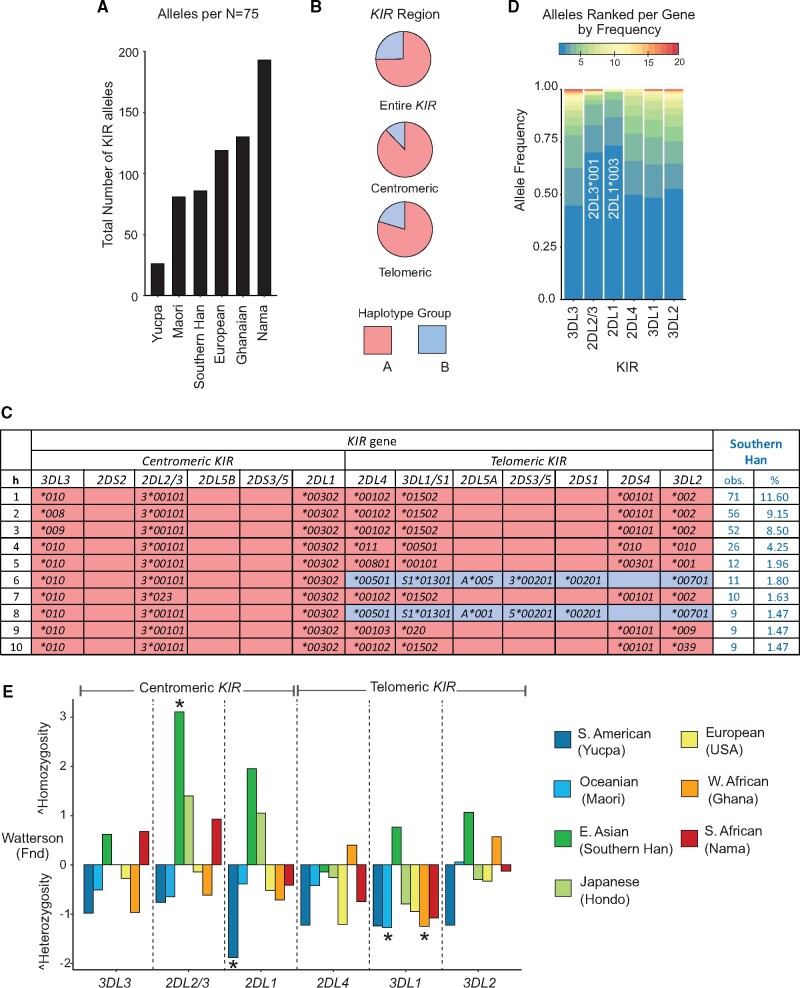
Directional selection on *centromeric KIR* in Southern Han. (*A*) Shows the number of *KIR* alleles present in the Southern Han compared with other populations analyzed at comparable resolution; 75 individuals were selected at random from each population. The Japanese group was not included because due to lack of *KIR3DL3* allele information. (*B*) Shown are the combined frequencies of *KIR A* (red) and *KIR B* (blue) haplotypes, for the complete haplotypes, and for the *centromeric* and *telomeric* regions. (*C*) Shown are the ten most frequent complete, high-resolution, *KIR* haplotypes identified in the Chinese Southern Han population. *KIR A* haplotypes are shaded in red, *KIR B* haplotypes are shaded in blue; the haplotypes are colored by segment. At the right is shown for each haplotype the number of individuals carrying the haplotype, and its frequency. All the haplotypes are shown in supplementary material S8 (Supplementary Material online). (*D*) Bar graph shows a summary *KIR* allele frequencies. The colors from blue to red correspond to the rank in frequency from highest (blue) to lowest (red). Full frequency distributions are shown in supplementary material S6 (Supplementary Material online). (*E*) Shown are normalized deviate values of Ewens–Watterson’s *F* test (F_nd_) in representative global populations. Positive values of F_nd_ indicate a deviation in homozygosity from that expected under neutrality, negative values indicate a deviation in heterozygosity from that expected under neutrality. An asterisk denotes significance (two-tailed *P*  < 0.05 or > 0.95) using the exact test (Salamon et al. 1999).

The *KIR* locus comprises genes encoding the four inhibitory and six activating KIR known to bind polymorphic HLA class I ligands and three that do not bind polymorphic HLA class I (Guethlein et al. 2015). In total, we identified 116 *KIR* alleles, representing 101 KIR allotypes (supplementary material S6, Supplementary Material online). A total of 46 novel *KIR* alleles (39.7% of total *KIR* alleles detected) were characterized (supplementary material S7, Supplementary Material online) and 24.8% of the individual Han carried at least one novel allele. Correcting for the number of individuals tested showed that the Southern Han are more diverse than Amerindians and Oceanians, but less diverse than Europeans and Africans (fig. 4*A*). *KIR* diversity of the CHS is thus consistent with genome-wide diversity when compared with the other populations (Campbell and Tishkoff 2008). The CHS have 70 centromeric and 91 telomeric *KIR* haplotype motifs that combine to form a minimum of 199 *KIR* haplotypes (supplementary material S8*A*–*C*, Supplementary Material online). The majority are *KIR A* haplotypes (74.7%, fig. 4*B*), including 8 of the 10 most frequent haplotypes (fig. 4*C*). This skewing toward *KIR A* haplotypes is more pronounced in the centromeric region (87.9%) than the telomeric (79.7%) region (figs. 4*C* and supplementary material S8*A–B*, Supplementary Material online).

*KIR A* haplotypes encode all four inhibitory receptors that bind HLA class I ligands and either one or no activating receptors (Wilson et al. 2000). Accordingly, among the *KIR* alleles identified in the CHS, we observed high frequencies of those encoding strong inhibitory receptors. Both KIR2DL1*003, a strongly inhibiting allotype of KIR2DL1 (Bari et al. 2009; Hilton et al. 2015a), and KIR2DL3*001, a strongly inhibiting allotype of KIR2DL2/3 (Yawata et al. 2006), are common in the CHS, having frequencies of 73.5% and 70.1%, respectively (supplementary material S6, Supplementary Material online). Also frequent are KIR3DL1*015, which is a strong inhibitor on binding to the Bw4 ligand (Yawata et al. 2006), and KIR3DL2*002 that has high expression but unknown functional properties (supplementary material S6, Supplementary Material online). Noticeably scarce are inhibitory KIR allotypes having mutations that prevent cell surface expression, of which there are many examples (Pando et al. 2003; VandenBussche et al. 2006; Bari et al. 2009; Hilton et al. 2015b). For instance, weakly expressed KIR3DL1*004 is present at allele frequencies of 5–15% in African, European, Oceanic, and South Asian populations (Norman et al. 2007; Nemat-Gorgani et al. 2014), but absent from the CHS [supplementary material S6, Supplementary Material online; Tao et al. (2014)]. Also rare in the CHS are alleles encoding inhibitory allotypes of reduced function, such as KIR2DL1*004 (3.6%, supplementary material S6, Supplementary Material online), which is widespread at 5–25% in African, European, Oceanic, and South Asian populations (Rajalingam et al. 2002; Meenagh et al. 2008; Bari et al. 2009; Vierra-Green et al. 2012; Norman et al. 2013; Nemat-Gorgani et al. 2014). Moreover, the frequencies of genes encoding activating receptors are much lower (4.4–18%) than those encoding inhibitory receptors (91.5–100%), an effect compounded by the presence of multiple nonfunctional activating KIR allotypes (supplementary material S6, Supplementary Material online). Exceptional is KIR2DS4, for which the frequencies of functional and nonfunctional allotypes are similar (55%:45%, supplementary material S6, Supplementary Material online). These observations point to a strong requirement in the Southern Han population for retaining high numbers of functional inhibitory KIR, but not activating KIR.

### Directional Selection Reduced Centromeric KIR Region Diversity in the Southern Han

In CHS, the *KIR3DL1/S1* and *KIR3DL2* genes encoding inhibitory NK cell receptors specific for polymorphic HLA class I ligands have two or three high frequency alleles and multiple less frequent alleles (supplementary material S6, Supplementary Material online). In contrast, *KIR2DL1* and *2DL2/3* also encode inhibitory receptors but are each dominated by one high frequency allele (fig. 4*D*). To explore this observation, we used the Ewens–Watterson test (*F*_nd_), which determines if homozygosity deviates within a population from that expected for the number of alleles present. We compared the observed homozygosity with the expected across populations representing major ancestry groups from Europe, Africa, Asia, South America, and Oceania. *KIR2DL1* and *KIR2DL2/3* are in the centromeric region of the *KIR* locus, whereas *KIR3DL1/S1* and *KIR3DL2* are telomeric *KIR* genes (Wilson et al. 2000). Overall, the Southern Han show greater deviation from expected homozygosity compared with other populations, and this is more pronounced among centromeric than telomeric *KIR* genes and statistically significant for *KIR2DL2/3* (F_nd_ = 3.1, two-tailed *P* < 0.05, fig. 4*E*). As the *F*_nd_ test is not corrected for demography, it does not allow us to make inferences as to whether the patterns we observed are due to demography or natural selection. However, our high-resolution analysis of KIR alleles complements a recent analysis of genome-wide SNP data that identified directional selection specifically in East Asian centromeric *KIR* (Augusto et al. 2019). Although not statistically significant here (fig. 4*E*), evidence for directional selection acting on the centromeric *KIR* region is also described for Hondo Japanese (Yawata et al. 2006). Thus, the profile observed for East Asian populations is distinct from other populations. Together, these independent analyses suggest that directional selection reduced sequence diversity of the centromeric *KIR* in the Southern Han, whereas the telomeric *KIR* region retains some diversity. In addition, we observed a minimum of eleven different *KIR* haplotypes having a duplication in the telomeric region (supplementary material S8D, Supplementary Material online). The telomeric *KIR* have greater allelic diversity than centromeric *KIR* in Chinese Han (supplementary material S8D, Supplementary Material online), and these duplication haplotypes have potential to further diversify the NK cell repertoire because both allotypes of each gene are expressed (Norman et al. 2009; Beziat et al. 2013). We conclude that the centromeric *KIR* region provides consistency to CHS NK cell functions, whereas the telomeric *KIR* region provides NK cell receptor diversity.

### Interactions of KIR with HLA Class I

**Fig. 5.**
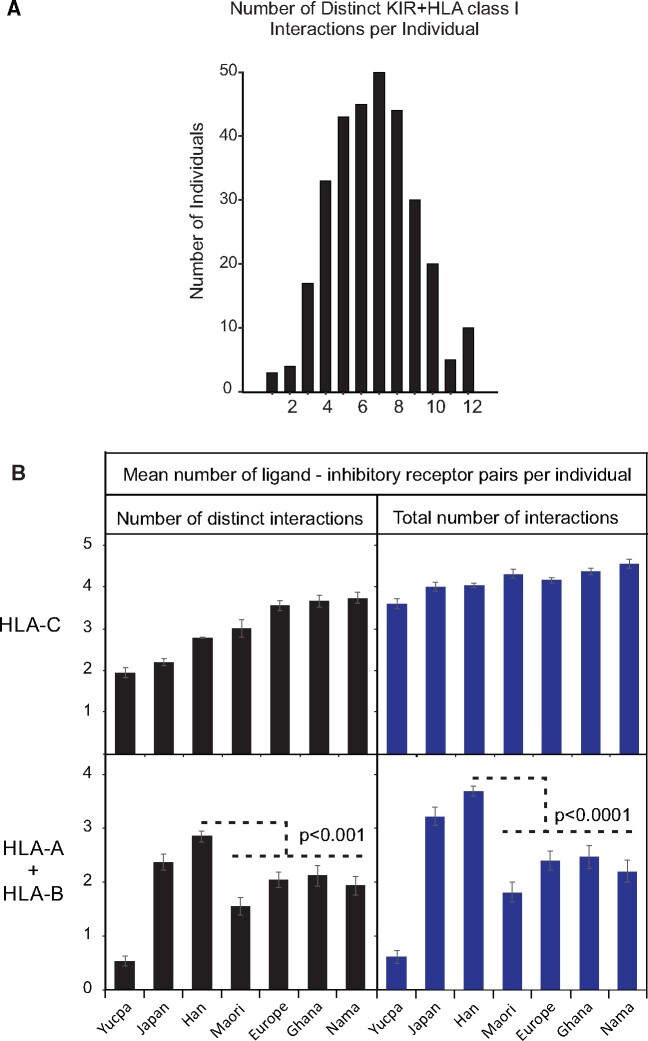
East Asians have a greater diversity of KIR interactions with HLA-A and -B than other populations. (*A*) Plot of the number of different interacting ligand/receptor allotype pairs observed per individual in the Southern Han. (*B*) Shows the mean number of different ligand/receptor allotype pairs per individual (left) and the mean total number of ligand/receptor allotype pairs per individual (right) for HLA-C (upper) and HLA-A and -B combined (lower). In the populations shown, *KIR* and *HLA class I* have been analyzed to similar high-resolution as described here for Southern Han. These populations comprise the Yucpa (Gendzekhadze et al. 2009), Japanese (Yawata et al. 2006), Māori (Nemat-Gorgani et al. 2014), European (Vierra-Green et al. 2012), Ghanaian (Norman et al. 2013) Nama (Nemat-Gorgani et al. 2018). Error bars are s.e.m. and p values are from a *t*-test.

NK cell function is modulated by interactions between KIR and their cognate ligands, HLA class I molecules. Whereas all HLA-C molecules are always ligands for KIR, only a subset of HLA-A and -B molecules function as KIR ligands. We examined the impact of genetic variation on the diversity and quantity of KIR/HLA class I interactions in the CHS. The studied individuals have a mean of 6.7 distinct pairs of interacting KIR and HLA class I ligands, forming a normal distribution from one to twelve interactions per individual (Shapiro–Wilk test, *P* = 0.147, fig. 5*A*). Such normal distributions are seen in other populations (Norman et al. 2013; Nemat-Gorgani et al. 2014). To investigate the distinct HLA-A and -B ligand distribution of the Southern Han, we divided this analysis into its major components of KIR interactions with HLA-C and of KIR interactions with HLA-A and -B (supplementary material S2, Supplementary Material online). In analyzing only the interactions with HLA-C, we find that functional diversity, as measured by the mean number of distinct receptor/ligand combinations per individual, is consistent with the overall genetic diversity of the populations studied. At the low end of the range are the Yucpa Amerindians with two distinct receptor/ligand interactions per individual. At the high end are the Southern African Nama with 4.5 different interactions (fig. 5*B*).

When the total number of interacting pairs of inhibitory KIR and HLA-C ligands is analyzed, the ranking remains the same, but the difference across populations is reduced, ranging from 3.6 to 4.9 viable inhibitory KIR/HLA-C interactions per individual (fig. 5*B*). On this scale, the CHS are seen to have relatively low diversity and a similar number of interactions between inhibitory KIR and HLA-C to other populations. In sharp contrast, the CHS, together with the Hondo Japanese, have significantly higher number (*t*-test, *P* < 0.0001) and diversity (*P* < 0.001), of inhibitory KIR interactions with HLA-A and -B than any other population (fig. 5*B*). Thus, both the quantity and strength of interactions between inhibitory KIR and HLA-A and -B are enhanced in Southern Han and Japanese. We predict that this will also be true for other East Asian populations.

## Discussion

Despite the significant differences in the distributions of *HLA class I* alleles across human populations (Meyer et al. 2007), the distribution of KIR ligands is very similar. In this context, our analysis identified an unusual distribution of HLA-A and -B-specific KIR ligands in CHS, representing the world’s largest population group. For *HLA-C*, all haplotypes encode one of two alternative KIR ligands, whereas for *HLA-A* and *HLA-B*, only subsets of allotypes carry KIR ligands; an example being the Bw4 motif. A potential reason is that HLA-C ligand sequences converge on a single ancestral origin, whereas the Bw4 motif can be shuffled among HLA-A or -B allotypes through meiotic recombination (Guethlein et al. 2015). Bw4 shows evidence for balancing selection across human populations, which maintain equivalent numbers of haplotypes that do or do not carry Bw4 (Norman et al. 2007). Accordingly, similar numbers of haplotypes with and without KIR ligands are attained independently in different human populations using very different HLA-A and -B allotypes. Due in part to high frequencies of the A3/11 ligand carried by subsets of HLA-A and C1 allotype carried by HLA-B*46, the Southern Han Chinese and other East Asian populations do not follow these patterns. We show that the number of KIR ligands at HLA-A and B has increased both directly through natural selection of allotypes that carry KIR ligands and indirectly through linkage with naturally selected alleles that are not ligands. The high frequencies of the *HLA class I* haplotypes shared by East Asian populations are an indication of their recent shared ancestry (Abdulla et al. 2009). We find a greater abundance of HLA-A and -B KIR ligands in East Asians than other populations as well as a greater diversity of interactions between inhibitory KIR and HLA-A and -B.

The frequency of East Asian *HLA class I* alleles that derive from ancient humans by introgression was previously estimated to be 70–80% (Abi-Rached et al. 2011). The most common of these alleles are HLA-A*11 and HLA-A*24, which encode KIR ligands. Previous studies have illustrated a complex series of demic dispersions in Central China with three waves moving from North to South in the past two millennia (Wen et al. 2004). This migration was followed by admixture events between the Han and South East Asian populations as well as a genetic differentiation occurring between Northern and Southern Han (Wen et al. 2004; Hellenthal et al. 2014). Consistent with these later admixture events, we show that HLA-B*46:01 and HLA-B*58:01, which also encode KIR ligands, were specifically enhanced in frequency in Southern Han. HLA-B*46 is a good educator of NK cells (Yawata et al. 2006) and rose in frequency in South East Asia under positive selection (Abi-Rached et al. 2010). The second most frequent haplotype, which encodes HLA-B*58, likely arose in Northern Asia, and although the signal is weaker, this may have been selected both in the Northern and Southern Han. Consequently, HLA-B*46:01 and HLA-B*58:01 are the most frequent *HLA-B* alleles encoding KIR ligands and distinguish the most frequent *HLA class I* haplotypes, in the Southern Han.

There is precedent in other modern human populations for adaptive introgression of *HLA* alleles (Rishishwar et al. 2015; Busby et al. 2017). For example, populations of Bantu speakers from western central Africa expanded through new habitats and acquired *HLA* haplotypes from rainforest hunter-gatherer pygmies (Patin et al. 2017). Multiple studies have identified that signals of recent admixture evident in Amerindian and Hispanic populations are enhanced in the *MHC* (Homburger et al. 2015; Rishishwar et al. 2015; Zhou et al. 2016; Meyer et al. 2018). A common theme of these studies is that the acquired HLA allotypes are beneficial for populations exposed to pathogens they had not previously encountered (Tang et al. 2007; Norris et al. 2020). Moreover, our findings may be consistent with recent work identifying a second wave of Denisovan-like admixture that is specific to East Asian populations (Browning et al. 2018). Thus, although we show that the HLA-B*46:01 and -B*58:01 haplotypes were obtained by the Han from neighboring modern human populations, they were likely to have been acquired by those populations as a consequence of admixture with archaic humans.

Complementing the high number of HLA class I ligands, we find that in the CHS, the number of inhibitory KIR is increased relative to other groups. These KIR allotypes are distinguished by their high expression, signal transduction strength and fine specificity for ligand (Hilton et al. 2015a; Saunders et al. 2016; Boudreau et al. 2017). Thus, whereas we do not provide direct evidence that selection to increase the number of KIR ligands is driving HLA distribution in East Asians, we show that many of the component parts of NK cell diversity have not evolved under neutrality. Strong and specific inhibition during NK cell education in the bone marrow enhances responsiveness of the mature NK cells to any loss of the respective HLA class I ligand that may occur during infection or tumorigenesis (Kim et al. 2005; Anfossi et al. 2006; Goodridge et al. 2019). Possessing higher numbers of inhibitory KIR thus leads to better effector function, and a higher number of inhibitory KIR ligands leads to larger numbers of circulating NK cells, stronger killing and greater diversity of the NK cell repertoire (Yawata et al. 2006; Brodin et al. 2009; Beziat et al. 2013). That the number of receptors (Pelak et al. 2011) and ligands (Thons et al. 2017) correlates with infection control, suggests the diverse NK cell repertoires of the Southern Han have likely evolved to combat infectious diseases common or endemic to East Asia. Although it is difficult to identify the specific pathogen exposure history of the CHS, the most plausible candidates for causing selection pressure are viral infections that have established roles for KIR/HLA interaction during host defense (Bashirova et al. 2006; Abi-Rached et al. 2010). Such pathogens have been shown to be effective drivers of adaptive introgression and natural selection in human populations (Enard and Petrov 2018; Harrison et al. 2019). One example is nasopharyngeal carcinoma (NPC) caused by Epstein–Barr virus. HLA-A*11 offers protection from NPC (Tang et al. 2012), and the interaction of KIR3DL2 with HLA-A*11 is dependent on presentation of peptides derived from EBV (Hansasuta et al. 2004). Influenza is another key candidate, with highly virulent epidemics linked to the combination of dense population, agriculture, and industrialization (Chen et al. 2006; Cao et al. 2009). Human-specific viral hepatitis infections and arboviruses are also endemic to China and South East Asia, including Japanese encephalitis, dengue, and chikungunya (Khakoo et al. 2004; Bashirova et al. 2006; Petitdemange et al. 2011; Townsley et al. 2016; Naiyer et al. 2017; Thons et al. 2017). Consistent with these observations, *KIR A* has established roles in controlling virus infections (Khakoo et al. 2004; Bashirova et al. 2006), and we recently showed that *KIR A* homozygosity protects from leukemia in CHS (Deng et al. 2019a). Reproduction is also a major driver of selection, where *KIR AA*/*C2^+^HLA-C* genotype is associated with increased risk for developing preeclampsia (Parham and Moffett 2013). Thus, the low frequency of C2^+^HLA in East Asia likely allows the *KIR A* haplotype to reach high frequency (Nemat-Gorgani et al. 2018). High resolution analysis of KIR and HLA diversity across East Asian populations (Wang et al. 2012; Bao et al. 2013; Yao et al. 2019) will be critical for understanding these and other complex diseases.

Genomic analyses and historical records have identified the Han migrated in three large waves from North to South during 265–316_AD,_ 618–907_AD_ and 1127–1279_AD_ (Ge et al. 1997; Wen et al. 2004; Stoneking and Delfin 2010). Concurrently, and separated by the Yangtze river, the Han became two genetically differentiated groups in the North and South. The significant admixture events between the CHS and South East Asians therefore likely occurred within the past 2,000 years. The timing of selection events for the endemic HLA-B*40:01 allele, and the admixed B*46:01 and B*58:01 alleles, can be speculated relative to these migrations, based on allele frequency, nucleotide diversity and |iHS| values. As expected, B*40:01 has a higher frequency in the Southern Han than the South East Asian populations (e.g. 0.23 and 0.08, respectively, for CHS and CDX [Abi-Rached et al. 2018)]. The haplotype carrying B*40:01 also has the highest nucleotide diversity and the lowest distribution of |iHS| values compared with B*58 and B*46, suggesting that this allele was under selection before the migration southward. Conversely, B*46:01 has a higher frequency in South East Asians than East Asians [0.32 and 0.17, respectively, for CDX and CHS (Abi-Rached et al. 2018)]. HLA-B*46:01 allele also has the lowest nucleotide diversity in the Southern Han and higher |iHS| values than B*40:01. Therefore, it is likely that the B*46:01 allele was beneficial to the Southern Han after migrating south. The frequency of B*58 is similar in East Asians and South East Asians. Of the 1,000 Genomes populations we analyzed, this allele has the highest frequency in the Beijing Han (0.08), which maintains more Northern Han ancestry than the Southern Han population does. Also, we identified a signature of Northern Han ancestry within carriers of the A*33:03-B*58:01 haplotype. Our findings are thus consistent with the previous suggestion that HLA-B*58:01 was obtained by admixture (Chen et al. 2016), and the likely Northern Han origin is further supported by the high frequency (7–20%) of HLA-B*58:01 in Northern Asia (Machulla et al. 2003). The nucleotide diversity of B*58 falls in a range between B*40 and B*46, and interestingly, distinct subsets of B*58 tagging SNPs exhibited |iHS| values that were all above the 99th percentile or all below the 95th percentile, which could indicate selection for standing variation.

In conclusion, our high-resolution analysis of KIR and HLA class I combinatorial diversity has uncovered a distinctive enhancement of the interactions between inhibitory KIR and HLA-A and -B in East Asians. These genetically determined distinctions likely underlie differences across human populations in their susceptibility to infections and immune-mediated diseases.

## Supplementary Material

Supplemental Data include five figures and three Excel spreadsheets.

## Supplementary Material

msab053_Supplementary_Data

## References

[msab053-B1] Abdulla MA Ahmed I Assawamakin A Bhak J Brahmachari SK Calacal GC Chaurasia A Chen C-H Chen J Chen Y-T , et al. 2009. Mapping Human Genetic Diversity in Asia. Science 326(5959):1541–1545. 20007900 10.1126/science.1177074

[msab053-B2] Abi-Rached L , Gouret P, Yeh JH, Di Cristofaro J, Pontarotti P, Picard C, Paganini J. 2018. Immune diversity sheds light on missing variation in worldwide genetic diversity panels. PLoS One 13(10):e0206512. 10.1371/journal.pone.0206512 PMC6203392 30365549

[msab053-B3] Abi-Rached L , Jobin MJ, Kulkarni S, McWhinnie A, Dalva K, Gragert L, Babrzadeh F, Gharizadeh B, Luo M, Plummer FA, et al 2011. The shaping of modern human immune systems by multiregional admixture with archaic humans. Science 334(6052):89–94. 21868630 10.1126/science.1209202 PMC3677943

[msab053-B4] Abi-Rached L , Moesta AK, Rajalingam R, Guethlein LA, Parham P. 2010. Human-specific evolution and adaptation led to major qualitative differences in the variable receptors of human and chimpanzee natural killer cells. PLoS Genet 6(11):e1001192. 21079681 10.1371/journal.pgen.1001192 PMC2973822

[msab053-B5] Alexander DH , Novembre J, Lange K. 2009. Fast model-based estimation of ancestry in unrelated individuals. Genome Res 19(9):1655–1664. 19648217 10.1101/gr.094052.109 PMC2752134

[msab053-B6] Andersson S , Fauriat C, Malmberg JA, Ljunggren HG, Malmberg KJ. 2009. KIR acquisition probabilities are independent of self-HLA class I ligands and increase with cellular KIR expression. Blood 114(1):95–104. 19304956 10.1182/blood-2008-10-184549

[msab053-B7] Anfossi N , Andre P, Guia S, Falk CS, Roetynck S, Stewart CA, Breso V, Frassati C, Reviron D, Middleton D, et al 2006. Human NK cell education by inhibitory receptors for MHC class I. Immunity 25(2):331–342. 16901727 10.1016/j.immuni.2006.06.013

[msab053-B8] Augusto DG , Norman PJ, Dandekar R, Hollenbach JA. 2019. Fluctuating and geographically specific selection characterize rapid evolution of the human *KIR* region. Front Immunol 10:989. 31156615 10.3389/fimmu.2019.00989 PMC6533848

[msab053-B9] Auton A , Brooks LD, Durbin RM, Garrison EP, Kang HM, Korbel JO, Marchini JL, McCarthy S, McVean GA, Abecasis GR, 2015. A global reference for human genetic variation. Nature 526(7571):68–74. 26432245 10.1038/nature15393 PMC4750478

[msab053-B10] Bao X , Wang M, Zhou H, Wu X, Yang L, Xu C, Yuan X, Zhang J, Li L, Wu D, et al 2013. Characterization of killer cell immunoglobulin-like receptor (KIR) genotypes and haplotypes in Chinese Han population. Tissue Antigens 82(5):327–337. 24131019 10.1111/tan.12211

[msab053-B11] Bao X , Wang M, Zhou H, Zhang H, Wu X, Yuan X, Li Y, Wu D, He J. 2016. Donor killer immunoglobulin-like receptor profile Bx1 imparts a negative effect and centromeric B-specific gene motifs render a positive effect on standard-risk acute myeloid leukemia/myelodysplastic syndrome patient survival after unrelated donor hematopoietic stem cell transplantation. Biol Blood Marrow Transpl 22(2):232–239. 10.1016/j.bbmt.2015.09.007 26371372

[msab053-B12] Bari R , Bell T, Leung WH, Vong QP, Chan WK, Das Gupta N, Holladay M, Rooney B, Leung W. 2009. Significant functional heterogeneity among KIR2DL1 alleles and a pivotal role of arginine 245. Blood 114(25):5182–5190. 19828694 10.1182/blood-2009-07-231977 PMC2792213

[msab053-B13] Bashirova AA , Martin MP, McVicar DW, Carrington M. 2006. The killer immunoglobulin-like receptor gene cluster: tuning the genome for defense. Annu Rev Genom Hum Genet 7(1):277–300. 10.1146/annurev.genom.7.080505.115726 16824023

[msab053-B14] Bastidas-Legarda LY , Khakoo SI. 2019. Conserved and variable natural killer cell receptors: diverse approaches to viral infections. Immunology 156(4):319–328. 30570753 10.1111/imm.13039 PMC6418463

[msab053-B15] Beziat V , Traherne JA, Liu LL, Jayaraman J, Enqvist M, Larsson S, Trowsdale J, Malmberg KJ. 2013. Influence of KIR gene copy number on natural killer cell education. Blood 121(23):4703–4707. 23637128 10.1182/blood-2012-10-461442 PMC3674669

[msab053-B16] Bjorkstrom NK , Ljunggren HG, Michaelsson J. 2016. Emerging insights into natural killer cells in human peripheral tissues. Nat Rev Immunol 16(5):310–320. 27121652 10.1038/nri.2016.34

[msab053-B17] Boelen L , Debebe B, Silveira M, Salam A, Makinde J, Roberts CH, Wang ECY, Frater J, Gilmour J, Twigger K, Ladell K, et al 2018. Inhibitory killer cell immunoglobulin-like receptors strengthen CD8^+^ T cell-mediated control of HIV-1, HCV, and HTLV-1. Sci Immunol 3:eaao2892. 10.1126/sciimmunol.aao2892 PMC6277004 30413420

[msab053-B18] Boudreau JE , Giglio F, Gooley TA, Stevenson PA, Le Luduec JB, Shaffer BC, Rajalingam R, Hou L, Hurley CK, Noreen H, et al 2017. KIR3DL1/HLA-B subtypes govern acute myelogenous leukemia relapse after hematopoietic cell transplantation. JCO 35(20):2268–2278. 10.1200/JCO.2016.70.7059 PMC5501362 28520526

[msab053-B19] Boudreau JE , Hsu KC. 2018. Natural killer cell education and the response to infection and cancer therapy: stay tuned. *Trends Immunol* 39:222–239 10.1016/j.it.2017.12.001 PMC6013060 29397297

[msab053-B20] Brodin P , Lakshmikanth T, Johansson S, Karre K, Hoglund P. 2009. The strength of inhibitory input during education quantitatively tunes the functional responsiveness of individual natural killer cells. Blood 113(11):2434–2441. 18974374 10.1182/blood-2008-05-156836

[msab053-B21] Browning SR , Browning BL, Zhou Y, Tucci S, Akey JM. 2018. Analysis of human sequence data reveals two pulses of archaic Denisovan admixture. Cell 173(1):53–61.e9. 29551270 10.1016/j.cell.2018.02.031 PMC5866234

[msab053-B22] Busby G , Christ R, Band G, Leffler E, Le QS, Rockett K, Kwiatkowski D, Spencer C. 2017. Inferring adaptive gene-flow in recent African history. *bioRxiv*. Forthcoming.

[msab053-B23] Campbell MC , Tishkoff SA. 2008. African genetic diversity: implications for human demographic history, modern human origins, and complex disease mapping. Annu Rev Genom Hum Genet 9(1):403–433. 10.1146/annurev.genom.9.081307.164258 PMC2953791 18593304

[msab053-B24] Cao B , Li XW, Mao Y, Wang J, Lu HZ, Chen YS, Liang ZA, Liang L, Zhang SJ, Zhang B, et al 2009. Clinical features of the initial cases of 2009 pandemic influenza A (H1N1) virus infection in China. N Engl J Med 361(26):2507–2517. 20007555 10.1056/NEJMoa0906612

[msab053-B25] Chen CH , Yang JH, Chiang CWK, Hsiung CN, Wu PE, Chang LC, Chu HW, Chang J, Song IW, Yang SL, Chen YT, et al 2016. Population structure of Han Chinese in the modern Taiwanese population based on 10,000 participants in the Taiwan Biobank project. Hum Mol Genet 25(24):5321–5331. 27798100 10.1093/hmg/ddw346 PMC6078601

[msab053-B26] Chen H , Smith GJ, Li KS, Wang J, Fan XH, Rayner JM, Vijaykrishna D, Zhang JX, Zhang LJ, Guo CT, Cheung CL, et al 2006. Establishment of multiple sublineages of H5N1 influenza virus in Asia: implications for pandemic control. Proc Natl Acad Sci USA 103(8):2845–2850. 16473931 10.1073/pnas.0511120103 PMC1413830

[msab053-B27] Cooper MA , Colonna M, Yokoyama WM. 2009. Hidden talents of natural killers: NK cells in innate and adaptive immunity. EMBO Rep 10(10):1103–1110. 19730434 10.1038/embor.2009.203 PMC2759738

[msab053-B28] Danecek P , Auton A, Abecasis G, Albers CA, Banks E, DePristo MA, Handsaker RE, Lunter G, Marth GT, Sherry ST, et al 2011. The variant call format and VCFtools. Bioinformatics 27(15):2156–2158. 21653522 10.1093/bioinformatics/btr330 PMC3137218

[msab053-B29] Dendrou CA , Petersen J, Rossjohn J, Fugger L. 2018. HLA variation and disease. Nat Rev Immunol 18(5):325–339. 29292391 10.1038/nri.2017.143

[msab053-B30] Deng Z , Zhao J, Cai S, Qi Y, Yu Q, Martin MP, Gao X, Chen R, Zhuo J, Zhen J, et al 2019a. Natural killer cells offer differential protection from leukemia in Chinese Southern Han. Front Immunol 10:1646. 31379844 10.3389/fimmu.2019.01646 PMC6646668

[msab053-B31] Deng Z , Zhen J, Zhang G. 2019b. Method for simultaneous sequence-based typing of 14 functional killer cell immunoglobulin-like receptor (KIR) genes. Patent 20180305744, USA.

[msab053-B32] Enard D , Petrov DA. 2018. Evidence that RNA viruses drove adaptive introgression between Neanderthals and modern humans. Cell 175(2):360–371.e13. 30290142 10.1016/j.cell.2018.08.034 PMC6176737

[msab053-B33] Excoffier L , Lischer HE. 2010. Arlequin suite ver 3.5: a new series of programs to perform population genetics analyses under Linux and Windows. Mol Ecol Resour 10(3):564–567. 21565059 10.1111/j.1755-0998.2010.02847.x

[msab053-B34] Fagundes NJ , Kanitz R, Eckert R, Valls AC, Bogo MR, Salzano FM, Smith DG, Silva WA Jr., Zago MA, Ribeiro-dos-Santos AK, et al 2008. Mitochondrial population genomics supports a single pre-Clovis origin with a coastal route for the peopling of the Americas. Am J Hum Genet 82(3):583–592. 18313026 10.1016/j.ajhg.2007.11.013 PMC2427228

[msab053-B35] Freud AG , Mundy-Bosse BL, Yu J, Caligiuri MA. 2017. The broad spectrum of human natural killer cell diversity. Immunity 47(5):820–833. 29166586 10.1016/j.immuni.2017.10.008 PMC5728700

[msab053-B36] Galili T. 2015. dendextend: an R package for visualizing, adjusting and comparing trees of hierarchical clustering. Bioinformatics 31(22):3718–3720. 26209431 10.1093/bioinformatics/btv428 PMC4817050

[msab053-B37] Ge J , Wu S, Chao S. 1997. Zhongguo Yimin Shi (The Migration History of China). Fuzhou (China): Fujian People’s Publishing House.

[msab053-B38] Gendzekhadze K , Norman PJ, Abi-Rached L, Graef T, Moesta AK, Layrisse Z, Parham P. 2009. Co-evolution of KIR2DL3 with HLA-C in a human population retaining minimal essential diversity of KIR and HLA class I ligands. Proc Natl Acad Sci USA 106(44):18692–18697. 19837691 10.1073/pnas.0906051106 PMC2774017

[msab053-B39] Gonzalez-Galarza FF , Takeshita LY, Santos EJ, Kempson F, Maia MH, da Silva AL, Teles e, Silva AL, Ghattaoraya GS, Alfirevic A, Jones AR, et al 2015. Allele frequency net 2015 update: new features for HLA epitopes, KIR and disease and HLA adverse drug reaction associations. Nucleic Acids Res 43(D1):D784–D788. 25414323 10.1093/nar/gku1166 PMC4383964

[msab053-B40] Goodridge JP , Jacobs B, Saetersmoen ML, Clement D, Hammer Q, Clancy T, Skarpen E, Brech A, Landskron J, Grimm C, et al 2019. Remodeling of secretory lysosomes during education tunes functional potential in NK cells. Nat Commun 10(1):514. 30705279 10.1038/s41467-019-08384-x PMC6355880

[msab053-B41] Gourraud PA , Khankhanian P, Cereb N, Yang SY, Feolo M, Maiers M, Rioux JD, Hauser S, Oksenberg J. 2014. HLA diversity in the 1000 genomes dataset. PLoS One 9(7):e97282. 24988075 10.1371/journal.pone.0097282 PMC4079705

[msab053-B42] Guethlein LA , Norman PJ, Hilton HH, Parham P. 2015. Co-evolution of MHC class I and variable NK cell receptors in placental mammals. Immunol Rev 267(1):259–282. 26284483 10.1111/imr.12326 PMC4587382

[msab053-B43] Hansasuta P , Dong T, Thananchai H, Weekes M, Willberg C, Aldemir H, Rowland-Jones S, Braud VM. 2004. Recognition of HLA-A3 and HLA-A11 by KIR3DL2 is peptide-specific. Eur J Immunol 34(6):1673–1679. 15162437 10.1002/eji.200425089

[msab053-B44] Harrison GF , Sanz J, Boulais J, Mina MJ, Grenier JC, Leng Y, Dumaine A, Yotova V, Bergey CM, Nsobya SL, et al 2019. Natural selection contributed to immunological differences between hunter-gatherers and agriculturalists. Nat Ecol Evol 3(8):1253–1264. 31358949 10.1038/s41559-019-0947-6 PMC6684323

[msab053-B45] Hellenthal G , Busby GBJ, Band G, Wilson JF, Capelli C, Falush D, Myers S. 2014. A genetic atlas of human admixture history. Science 343(6172):747–751. 24531965 10.1126/science.1243518 PMC4209567

[msab053-B46] Henn BM , Cavalli-Sforza LL, Feldman MW. 2012. The great human expansion. Proc Natl Acad Sci USA 109(44):17758–17764. 23077256 10.1073/pnas.1212380109 PMC3497766

[msab053-B47] Hilton HG , Guethlein LA, Goyos A, Nemat-Gorgani N, Bushnell DA, Norman PJ, Parham P. 2015a. Polymorphic HLA-C receptors balance the functional characteristics of KIR haplotypes. J Immunol 195(7):3160–3170. 26311903 10.4049/jimmunol.1501358 PMC4575877

[msab053-B48] Hilton HG , Norman PJ, Nemat-Gorgani N, Goyos A, Hollenbach JA, Henn BM, Gignoux CR, Guethlein LA, Parham P. 2015b. Loss and gain of natural killer cell receptor function in an African hunter-gatherer population. PLoS Genet 11(8):e1005439. 26292085 10.1371/journal.pgen.1005439 PMC4546388

[msab053-B49] Hoglund P , Brodin P. 2010. Current perspectives of natural killer cell education by MHC class I molecules. Nat Rev Immunol 10(10):724–734. 20818413 10.1038/nri2835

[msab053-B50] Holzemer A , Garcia-Beltran WF, Altfeld M. 2017. Natural killer cell interactions with classical and non-classical human leukocyte antigen class I in HIV-1 infection. Front Immunol 8:1496. 29184550 10.3389/fimmu.2017.01496 PMC5694438

[msab053-B51] Homburger JR , Moreno-Estrada A, Gignoux CR, Nelson D, Sanchez E, Ortiz-Tello P, Pons-Estel BA, Acevedo-Vasquez E, Miranda P, Langefeld CD, et al 2015. Genomic insights into the ancestry and demographic history of South America. PLoS Genet 11(12):e1005602. 26636962 10.1371/journal.pgen.1005602 PMC4670080

[msab053-B52] Jiang Y , Chen O, Cui C, Zhao B, Han X, Zhang Z, Liu J, Xu J, Hu Q, Liao C, et al 2013. KIR3DS1/L1 and HLA-Bw4-80I are associated with HIV disease progression among HIV typical progressors and long-term nonprogressors. BMC Infect Dis 13(1):405. 24059286 10.1186/1471-2334-13-405 PMC3766012

[msab053-B53] Khakoo SI , Thio CL, Martin MP, Brooks CR, Gao X, Astemborski J, Cheng J, Goedert JJ, Vlahov D, Hilgartner M, et al 2004. HLA and NK cell inhibitory receptor genes in resolving hepatitis C virus infection. Science 305(5685):872–874. 15297676 10.1126/science.1097670

[msab053-B54] Kim S , Poursine-Laurent J, Truscott SM, Lybarger L, Song YJ, Yang L, French AR, Sunwoo JB, Lemieux S, Hansen TH, et al 2005. Licensing of natural killer cells by host major histocompatibility complex class I molecules. Nature 436(7051):709–713. 16079848 10.1038/nature03847

[msab053-B55] Lancaster AK , Single RM, Solberg OD, Nelson MP, Thomson G. 2007. PyPop update—a software pipeline for large-scale multilocus population genomics. Tissue Antigens 69:192–197. 17445199 10.1111/j.1399-0039.2006.00769.x PMC4369784

[msab053-B56] Loh P-R , Palamara PF, Price AL. 2016. Fast and accurate long-range phasing in a UK Biobank cohort. Nat Genet 48(7):811–816. 27270109 10.1038/ng.3571 PMC4925291

[msab053-B57] Long EO , Kim HS, Liu D, Peterson ME, Rajagopalan S. 2013. Controlling natural killer cell responses: integration of signals for activation and inhibition. Annu Rev Immunol 31(1):227–258. 23516982 10.1146/annurev-immunol-020711-075005 PMC3868343

[msab053-B58] Long W , Shi Z, Fan S, Liu L, Lu Y, Guo X, Rong C, Cui X, Ding H. 2015. Association of maternal *KIR* and fetal *HLA-C* genes with the risk of preeclampsia in the Chinese Han population. Placenta 36(4):433–437. 24951171 10.1016/j.placenta.2014.05.008

[msab053-B59] Lu D , Lou H, Yuan K, Wang X, Wang Y, Zhang C, Lu Y, Yang X, Deng L, Zhou Y, et al 2016. Ancestral origins and genetic history of Tibetan Highlanders. Am J Hum Genet 99(3):580–594. 27569548 10.1016/j.ajhg.2016.07.002 PMC5011065

[msab053-B60] Machulla HK , Batnasan D, Steinborn F, Uyar FA, Saruhan-Direskeneli G, Oguz FS, Carin MN, Dorak MT. 2003. Genetic affinities among Mongol ethnic groups and their relationship to Turks. Tissue Antigens 61(4):292–299. 12753667 10.1034/j.1399-0039.2003.00043.x

[msab053-B61] Meenagh A , Gonzalez A, Sleator C, McQuaid S, Middleton D. 2008. Investigation of killer cell immunoglobulin-like receptor gene diversity, *KIR2DL1* and *KIR2DS1>*. Tissue Antigens 72(4):383–391. 18643963 10.1111/j.1399-0039.2008.01093.x

[msab053-B62] Meyer D , Aguiar VR, Bitarello BD, Brandt DY, Nunes K. 2018. A genomic perspective on HLA evolution. Immunogenetics 70(1):5–27. 28687858 10.1007/s00251-017-1017-3 PMC5748415

[msab053-B63] Meyer D , Single RM, Mack SJ, Lancaster A, Nelson MP, Erlich HA, Fernandez-Vina M, Thomson G. 2007. Single locus polymorphism of classical *HLA* genes. In: Hansen JA (ed) Immunobiology of the Human MHC. Proceedings of the 13th International Histocompatibility Workshop and Conference. Seattle (WA): IHWG Press.

[msab053-B64] Naiyer MM , Cassidy SA, Magri A, Cowton V, Chen K, Mansour S, Kranidioti H, Mbirbindi B, Rettman P, Harris S, et al 2017. KIR2DS2 recognizes conserved peptides derived from viral helicases in the context of HLA-C. *Sci Immunol* 2:eaa15296. 10.1126/sciimmunol.aal5296 28916719

[msab053-B65] Nei M , Takahata N. 1993. Effective population size, genetic diversity, and coalescence time in subdivided populations. J Mol Evol 37(3):240–244. 8230248 10.1007/BF00175500

[msab053-B66] Nemat-Gorgani N , Edinur HA, Hollenbach JA, Traherne JA, Dunn PP, Chambers GK, Parham P, Norman PJ. 2014. KIR diversity in Maori and Polynesians: populations in which HLA-B is not a significant KIR ligand. Immunogenetics. 66(11):597–611. 25139336 10.1007/s00251-014-0794-1 PMC4198482

[msab053-B67] Nemat-Gorgani N , Hilton HG, Henn BM Lin M, Gignoux CR, Myrick JW, Werely CJ, Granka JM, Moller M, Hoal EG, Yawata M, et al 2018. Different selected mechanisms attenuated the inhibitory interaction of KIR2DL1 with C2^+^ HLA-C in two indigenous human populations in Southern Africa. *J Immunol* 200:2640–2655. 10.4049/jimmunol.1701780 PMC5990024 29549179

[msab053-B68] Norman PJ , Abi-Rached L, Gendzekhadze K, Hammond JA, Moesta AK, Sharma D, Graef T, McQueen KL, Guethlein LA, Carrington CV, et al 2009. Meiotic recombination generates rich diversity in NK cell receptor genes, alleles, and haplotypes. Genome Res 19(5):757–769. 19411600 10.1101/gr.085738.108 PMC2675964

[msab053-B69] Norman PJ , Abi-Rached L, Gendzekhadze K, Korbel D, Gleimer M, Rowley D, Bruno D, Carrington CV, Chandanayingyong D, Chang YH, et al 2007. Unusual selection on the KIR3DL1/S1 natural killer cell receptor in Africans. Nat Genet 39(9):1092–1099. 17694054 10.1038/ng2111

[msab053-B70] Norman PJ , Hollenbach JA, Nemat-Gorgani N, Guethlein LA, Hilton HG, Pando MJ, Koram KA, Riley EM, Abi-Rached L, Parham P. 2013. Co-evolution of human leukocyte antigen (HLA) class I ligands with killer-cell immunoglobulin-like receptors (KIR) in a genetically diverse population of Sub-Saharan Africans. PLoS Genet 9(10):e1003938. 24204327 10.1371/journal.pgen.1003938 PMC3814319

[msab053-B71] Norman PJ , Hollenbach JA, Nemat-Gorgani N, Marin WM, Norberg SJ, Ashouri E, Jayaraman J, Wroblewski EE, Trowsdale J, Rajalingam R, Oksenberg JR, Chiaroni J, et al 2016. Defining KIR and HLA class I genotypes at highest resolution via high-throughput sequencing. Am J Hum Genet 99(2):375–391. 27486779 10.1016/j.ajhg.2016.06.023 PMC4974113

[msab053-B72] Norris ET , Rishishwar L, Chande AT, Conley AB, Ye K, Valderrama-Aguirre A, Jordan IK. 2020. Admixture-enabled selection for rapid adaptive evolution in the Americas. Genome Biol 21(1):1–12. 10.1186/s13059-020-1946-2 PMC7006128 32028992

[msab053-B73] Pando MJ , Gardiner CM, Gleimer M, McQueen KL, Parham P. 2003. The protein made from a common allele of KIR3DL1 (3DL1004) is poorly expressed at cell surfaces due to substitution at positions 86 in Ig domain 0 and 182 in Ig domain 1. J Immunol 171(12):6640–6649. 14662867 10.4049/jimmunol.171.12.6640

[msab053-B74] Parham P , Moffett A. 2013. Variable NK cell receptors and their MHC class I ligands in immunity, reproduction and human evolution. Nat Rev Immunol 13(2):133–144. 23334245 10.1038/nri3370 PMC3956658

[msab053-B75] Patin E , Lopez M, Grollemund R, Verdu P, Harmant C, Quach H, Laval G, Perry GH, Barreiro LB, Froment A, et al 2017. Dispersals and genetic adaptation of Bantu-speaking populations in Africa and North America. Science 356(6337):543–546. 28473590 10.1126/science.aal1988

[msab053-B76] Pelak K , Need AC, Fellay J, Shianna KV, Feng S, Urban TJ, Ge D, De Luca A, Martinez-Picado J, Wolinsky SM, et al 2011. Copy number variation of KIR genes influences HIV-1 control. PLoS Biol 9(11):e1001208., 22140359 10.1371/journal.pbio.1001208 PMC3226550

[msab053-B77] Petitdemange C , Becquart P, Wauquier N, Beziat V, Debre P, Leroy EM, Vieillard V. 2011. Unconventional repertoire profile is imprinted during acute chikungunya infection for natural killer cells polarization toward cytotoxicity. PLoS Pathog 7(9):e1002268. 21966274 10.1371/journal.ppat.1002268 PMC3178577

[msab053-B78] Prugnolle F , Manica A, Charpentier M, Guégan JF, Guernier V, Balloux F. 2005. Pathogen-driven selection and worldwide HLA class I diversity. Curr Biol 15(11):1022–1027. 15936272 10.1016/j.cub.2005.04.050

[msab053-B79] Purcell S , Neale B, Todd-Brown K, Thomas L, Ferreira MA, Bender D, Maller J, Sklar P, de Bakker PI, Daly MJ, et al 2007. PLINK: a tool set for whole-genome association and population-based linkage analyses. Am J Hum Genet 81(3):559–575. 17701901 10.1086/519795 PMC1950838

[msab053-B80] Development Core Team R. 2008. A language and environment for statistical computing. Vienna ( Austria): R Foundation for Statistical Computing.

[msab053-B81] Raghavan M , Steinrucken M, Harris K, Schiffels S, Rasmussen S, DeGiorgio M, Albrechtsen A, Valdiosera C, Avila-Arcos MC, Malaspinas AS, et al 2015. Population genetics. Genomic evidence for the Pleistocene and recent population history of Native Americans. Science 349(6250):aab3884–aab3884. 26198033 10.1126/science.aab3884 PMC4733658

[msab053-B82] Rajalingam R , Krausa P, Shilling HG, Stein JB, Balamurugan A, McGinnis MD, Cheng NW, Mehra NK, Parham P. 2002. Distinctive KIR and HLA diversity in a panel of north Indian Hindus. Immunogenetics 53(12):1009–1019. 11904677 10.1007/s00251-001-0425-5

[msab053-B83] Rishishwar L , Conley AB, Wigington CH, Wang L, Valderrama-Aguirre A, Jordan IK. 2015. Ancestry, admixture and fitness in Colombian genomes. Sci Rep 5(1):12376. 26197429 10.1038/srep12376 PMC4508918

[msab053-B84] Robinson J , Halliwell JA, Hayhurst JD, Flicek P, Parham P, Marsh SG. 2015. The IPD and IMGT/HLA database: allele variant databases. Nucleic Acids Res 43(D1):D423–D431. 25414341 10.1093/nar/gku1161 PMC4383959

[msab053-B85] Rosenberg NA , Pritchard JK, Weber JL, Cann HM, Kidd KK, Zhivotovsky LA, Feldman MW. 2002. Genetic structure of human populations. Science 298(5602):2381–2385. 12493913 10.1126/science.1078311

[msab053-B86] Salamon H , Klitz W, Easteal S, Gao X, Erlich HA, Fernandez-Vina M, Trachtenberg EA, McWeeney SK, Nelson MP, Thomson G. 1999. Evolution of HLA class II molecules: allelic and amino acid site variability across populations. Genetics 152:393–400. 10224269 10.1093/genetics/152.1.393 PMC1460587

[msab053-B87] Saunders PM , Pymm P, Pietra G, Hughes VA, Hitchen C, O’Connor GM, Loiacono F, Widjaja J, Price DA, Falco M, et al 2016. Killer cell immunoglobulin-like receptor 3DL1 polymorphism defines distinct hierarchies of HLA class I recognition. J Exp Med 213(5):791–807. 27045007 10.1084/jem.20152023 PMC4854737

[msab053-B88] Saunders PM , Vivian JP, O’Connor GM, Sullivan LC, Pymm P, Rossjohn J, Brooks AG. 2015. A bird’s eye view of NK cell receptor interactions with their MHC class I ligands. Immunol Rev 267(1):148–166. 26284476 10.1111/imr.12319

[msab053-B89] Schiffels S , Durbin R. 2014. Inferring human population size and separation history from multiple genome sequences. Nat Genet 46(8):919–925. 24952747 10.1038/ng.3015 PMC4116295

[msab053-B90] Shen M , Linn YC, Ren EC. 2016. KIR-HLA profiling shows presence of higher frequencies of strong inhibitory KIR-ligands among prognostically poor risk AML patients. Immunogenetics 68(2):133–144. 26649563 10.1007/s00251-015-0888-4

[msab053-B91] Solberg OD , Mack SJ, Lancaster AK, Single RM, Tsai Y, Sanchez-Mazas A, Thomson G. 2008. Balancing selection and heterogeneity across the classical human leukocyte antigen loci: a meta-analytic review of 497 population studies. Hum Immunol 69(7):443–464. 18638659 10.1016/j.humimm.2008.05.001 PMC2632948

[msab053-B92] Stephens M , Donnelly P. 2003. A comparison of bayesian methods for haplotype reconstruction from population genotype data. Am J Hum Genet 73(5):1162–1169. 14574645 10.1086/379378 PMC1180495

[msab053-B93] Stoneking M , Delfin F. 2010. The human genetic history of East Asia: weaving a complex tapestry. Curr Biol 20(4):R188–R193. 20178766 10.1016/j.cub.2009.11.052

[msab053-B94] Su N , Wang H, Zhang B, Kang Y, Guo Q, Xiao H, Yang H, Liao S. 2018. Maternal natural killer cell immunoglobulin receptor genes and human leukocyte antigen-C ligands influence recurrent spontaneous abortion in the Han Chinese population. Exp Ther Med 15:327–337. 29387191 10.3892/etm.2017.5406 PMC5769230

[msab053-B95] Szpiech ZA , Hernandez RD. 2014. selscan: an efficient multithreaded program to perform EHH-based scans for positive selection. Mol Biol Evol 31(10):2824–2827. 25015648 10.1093/molbev/msu211 PMC4166924

[msab053-B96] Takeuchi F , Katsuya T, Kimura R, Nabika T, Isomura M, Ohkubo T, Tabara Y, Yamamoto K, Yokota M, Liu X, et al 2017. The fine-scale genetic structure and evolution of the Japanese population. PLoS One 12(11):e0185487. 29091727 10.1371/journal.pone.0185487 PMC5665431

[msab053-B97] Tang H , Choudhry S, Mei R, Morgan M, Rodriguez-Cintron W, Burchard EG, Risch NJ. 2007. Recent genetic selection in the ancestral admixture of Puerto Ricans. Am J Hum Genet 81(3):626–633. 17701908 10.1086/520769 PMC1950843

[msab053-B98] Tang M , Lautenberger JA, Gao X, Sezgin E, Hendrickson SL, Troyer JL, David VA, Guan L, McIntosh CE, Guo X, et al 2012. The principal genetic determinants for nasopharyngeal carcinoma in China involve the HLA class I antigen recognition groove. PLoS Genet 8(11):e1003103. 23209447 10.1371/journal.pgen.1003103 PMC3510037

[msab053-B99] Tao SD , He YM, Ying YL, He J, Zhu FM, Lv HJ. 2014. KIR3DL1 genetic diversity and phenotypic variation in the Chinese Han population. Genes Immun 15(1):8–15. 24173144 10.1038/gene.2013.55

[msab053-B100] Thons C , Senff T, Hydes TJ, Manser AR, Heinemann FM, Heinold A, Heilmann M, Kim AY, Uhrberg M, Scherbaum N, et al 2017. HLA-Bw4 80(T) and multiple HLA-Bw4 copies combined with KIR3DL1 associate with spontaneous clearance of HCV infection in people who inject drugs. J Hepatol 67(3):462–470. 28412292 10.1016/j.jhep.2017.03.040

[msab053-B101] Tishkoff SA , Reed FA, Friedlaender FR, Ehret C, Ranciaro A, Froment A, Hirbo JB, Awomoyi AA, Bodo JM, Doumbo O, et al 2009. The genetic structure and history of Africans and African Americans. Science 324(5930):1035–1044. 19407144 10.1126/science.1172257 PMC2947357

[msab053-B102] Townsley E , O’Connor G, Cosgrove C, Woda M, Co M, Thomas SJ, Kalayanarooj S, Yoon IK, Nisalak A, Srikiatkhachorn A, et al 2016. Interaction of a dengue virus NS1-derived peptide with the inhibitory receptor KIR3DL1 on natural killer cells. Clin Exp Immunol 183(3):419–430. 26439909 10.1111/cei.12722 PMC4750593

[msab053-B103] VandenBussche CJ , Dakshanamurthy S, Posch PE, Hurley CK. 2006. A single polymorphism disrupts the killer Ig-like receptor 2DL2/2DL3 D1 domain. J Immunol 177(8):5347–5357. 17015720 10.4049/jimmunol.177.8.5347

[msab053-B104] Vierra-Green C , Roe D, Hou L, Hurley CK, Rajalingam R, Reed E, Lebedeva T, Yu N, Stewart M, Noreen H, et al 2012. Allele-level haplotype frequencies and pairwise linkage disequilibrium for 14 *KIR* loci in 506 European-American individuals. PLoS One 7(11):e47491. 23139747 10.1371/journal.pone.0047491 PMC3489906

[msab053-B105] Vivier E , Raulet DH, Moretta A, Caligiuri MA, Zitvogel L, Lanier LL, Yokoyama WM, Ugolini S. 2011. Innate or adaptive immunity? The example of natural killer cells. Science 331(6013):44–49. 21212348 10.1126/science.1198687 PMC3089969

[msab053-B106] Voight BF , Kudaravalli S, Wen X, Pritchard JK. 2006. A map of recent positive selection in the human genome. PLoS Biol 4(3):e72. 16494531 10.1371/journal.pbio.0040072 PMC1382018

[msab053-B107] Wang HD , Zhang FX, Shen CM, Wu YM, Lv YG, Xie ST, Yang G, Qin HX, Fan SL, Zhu BF. 2012. The distribution of genetic diversity of *KIR* genes in the Chinese Mongolian population. Hum Immunol 73(10):1031–1038. 22836038 10.1016/j.humimm.2012.07.317

[msab053-B108] Wen B , Li H, Lu D, Song X, Zhang F, He Y, Li F, Gao Y, Mao X, Zhang L, et al 2004. Genetic evidence supports demic diffusion of Han culture. Nature 431(7006):302–305. 15372031 10.1038/nature02878

[msab053-B109] Wilson MJ , Torkar M, Haude A, Milne S, Jones T, Sheer D, Beck S, Trowsdale J. 2000. Plasticity in the organization and sequences of human *KIR*/*ILT* gene families. Proc Natl Acad Sci USA 97(9):4778–4783. 10781084 10.1073/pnas.080588597 PMC18309

[msab053-B110] Xu S , Yin X, Li S, Jin W, Lou H, Yang L, Gong X, Wang H, Shen Y, Pan X, et al 2009. Genomic dissection of population substructure of Han Chinese and its implication in association studies. Am J Hum Genet 85(6):762–774. 19944404 10.1016/j.ajhg.2009.10.015 PMC2790582

[msab053-B111] Yao Y , Shi L, Tao Y, Lin K, Liu S, Yu L, Yang Z, Yi W, Huang X, Sun H, et al 2011. Diversity of killer cell immunoglobulin-like receptor genes in four ethnic groups in China. Immunogenetics 63(8):475–483. 21556858 10.1007/s00251-011-0530-z

[msab053-B112] Yao Y , Shi L, Yu J, Liu S, Tao Y, Shi L. 2019. Distribution of killer-cell immunoglobulin-like receptor genes and combinations of their human leucocyte antigen ligands in 11 ethnic populations in China. Cell 8(7):711. 10.3390/cells8070711 PMC6678321 31336930

[msab053-B113] Yawata M , Yawata N, Draghi M, Little AM, Partheniou F, Parham P. 2006. Roles for HLA and KIR polymorphisms in natural killer cell repertoire selection and modulation of effector function. J Exp Med 203(3):633–645. 16533882 10.1084/jem.20051884 PMC2118260

[msab053-B114] Zhang G , Deng Z. 2016. [A strategy to clarify ambiguities during genotyping of functional KIR framework genes by sequencing-based typing among ethnic Hans from southern China]. Zhonghua Yi Xue Yi Chuan Xue Za Zhi 33(6):773–777. 27984603 10.3760/cma.j.issn.1003-9406.2016.06.006

[msab053-B115] Zhou Q , Zhao L, Guan Y. 2016. Strong selection at MHC in Mexicans since admixture. PLoS Genet 12(2):e1005847. 26863142 10.1371/journal.pgen.1005847 PMC4749250

