# Supplementary material for: Adaptive Admixture of HLA Class I Allotypes Enhanced Genetically Determined Strength of Natural Killer Cells in East Asians
Source: Mol Biol Evol. 2021 Feb 22;38(6):2582–96. doi: 10.1093/molbev/msab053 (PMC8136484; doi:10.1093/molbev/msab053)
Supplement: msab053_Supplementary_Data [file msab053_supplementary_data.zip › Hans_paper_Supps.pdf]

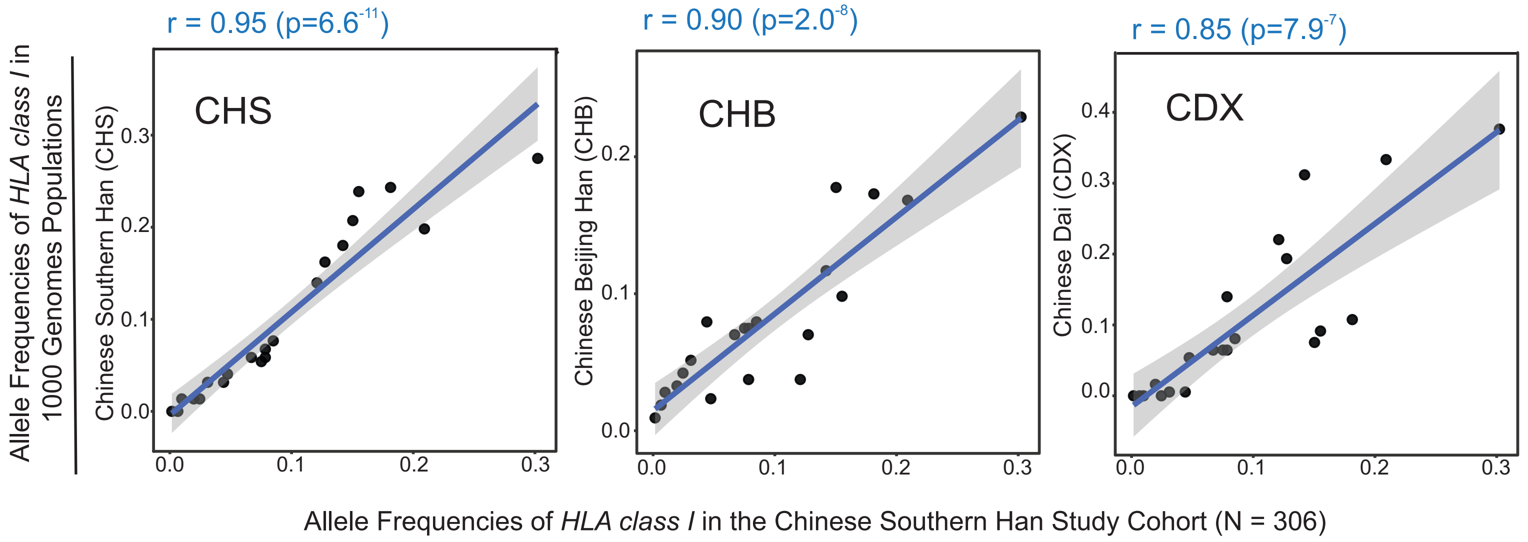

r = 0.95 (p=6.6-11)
r = 0.90 (p=2.0-8)
r = 0.85 (p=7.9-7)
Allele Frequencies of HLA class I in 1000 Genomes Populations
CHS
CHB
CDX
Chinese Southern Han (CHS)
Chinese Beijing Han (CHB)
Chinese Dai (CDX)
0.0
0.1
0.2
0.3
0.4
Allele Frequencies of HLA class I in the Chinese Southern Han Study Cohort (N = 306)

Figure S1

HLA-A		HLA-B			HLA-C	
Allele	Frequency (%)	Allele	-21M/T	Frequency (%)	Allele	Frequency (%)
*A*01:01*	2.45	*B*07:02*	M	0.98	*C*01:02*	20.92
*A*01:03*	0.16	*B*07:05*	M	0.49	*C*01:03*	0.33
*A*02:01*	8.82	*B*08:01*	M	0.65	*C*02:02*	0.16
*A*02:03*	5.39	*B*13:01*	T	7.84	*C*03:01*	0.16
*A*02:05*	0.33	*B*13:02*	T	2.12	*C*03:02*	7.52
*A*02:06*	2.45	*B*14:02*	M	0.16	*C*03:03*	4.58
*A*02:07*	12.26	*B*15:01*	T	3.6	*C*03:04*	12.75
*A*02:17*	0.16	*B*15:02*	T	4.74	*C*03:17*	0.16
*A*03:01*	1.14	*B*15:05*	T	0.49	*C*03:38*	0.16
*A*03:02*	0.16	*B*15:07*	T	0.16	*C*04:01*	4.58
*A*11:01*	30.23	*B*15:11*	T	1.14	*C*04:03*	1.8
*A*11:02*	2.78	*B*15:12*	T	0.49	*C*05:01*	0.49
*A*11:03*	0.16	*B*15:18*	T	0.65	*C*06:02*	4.41
*A*11:188*	0.16	*B*15:21*	T	0.16	*C*07:01*	0.49
*A*23:01*	0.33	*B*15:25*	T	0.49	*C*07:02*	18.14
*A*24:02*	15.19	*B*15:27*	T	0.33	*C*07:04*	0.65
*A*24:10*	0.16	*B*18:01*	T	0.33	*C*07:06*	0.33
*A*26:01*	1.96	*B*27:04*	T	1.31	*C*07:19*	0.49
*A*29:01*	0.65	*B*27:05*	T	0.16	*C*07:43*	0.16
*A*30:01*	2.45	*B*27:06*	T	0.16	*C*08:01*	8.5
*A*31:01*	3.11	*B*35:01*	T	2.29	*C*08:02*	0.16
*A*32:01*	1.47	*B*35:02*	T	0.16	*C*08:03*	0.49
*A*33:01*	0.33	*B*35:03*	T	0.33	*C*12:02*	2.94
*A*33:03*	6.86	*B*37:01*	T	1.14	*C*12:03*	2.12
*A*34:01*	0.16	*B*38:01*	M	0.16	*C*14:02*	3.43
*A*68:01*	0.49	*B*38:02*	M	2.94	*C*14:03*	0.16
*A*74:02*	0.16	*B*39:01*	M	1.96	*C*15:02*	3.11
		*B*39:05*	M	0.16	*C*15:05*	0.49
		*B*40:01*	T	15.52	*C*16:02*	0.16
		*B*40:02*	T	1.31	*C*17:01*	0.16
		*B*40:06*	T	1.96		
		*B*40:40*	T	0.16		
		*B*41:01*	T	0.16		
		*B*44:02*	T	0.65		
		*B*44:03*	T	0.65		
		*B*44:07*	T	0.16		
		*B*46:01*	T	14.22		
		*B*48:01*	M	0.98		
		*B*48:03*	M	0.82		
		*B*49:01*	T	0.16		
		*B*50:01*	T	0.16		
		*B*51:01*	T	3.76		
		*B*51:02*	T	1.31		
		*B*52:01*	T	2.12		
		*B*54:01*	T	4.09		
		*B*55:01*	T	0.16		
		*B*55:02*	T	4.09		
		*B*56:01*	T	1.63		
		*B*56:04*	T	0.16		
		*B*57:01*	T	1.14		
		*B*58:01*	T	7.84		
		*B*59:01*	T	0.16		
		*B*67:01*	M	0.65		
		*B*81:02*	M	0.33		

KIR ligand

- A3/11
- Bw4
- C1
- C2

Figure S3

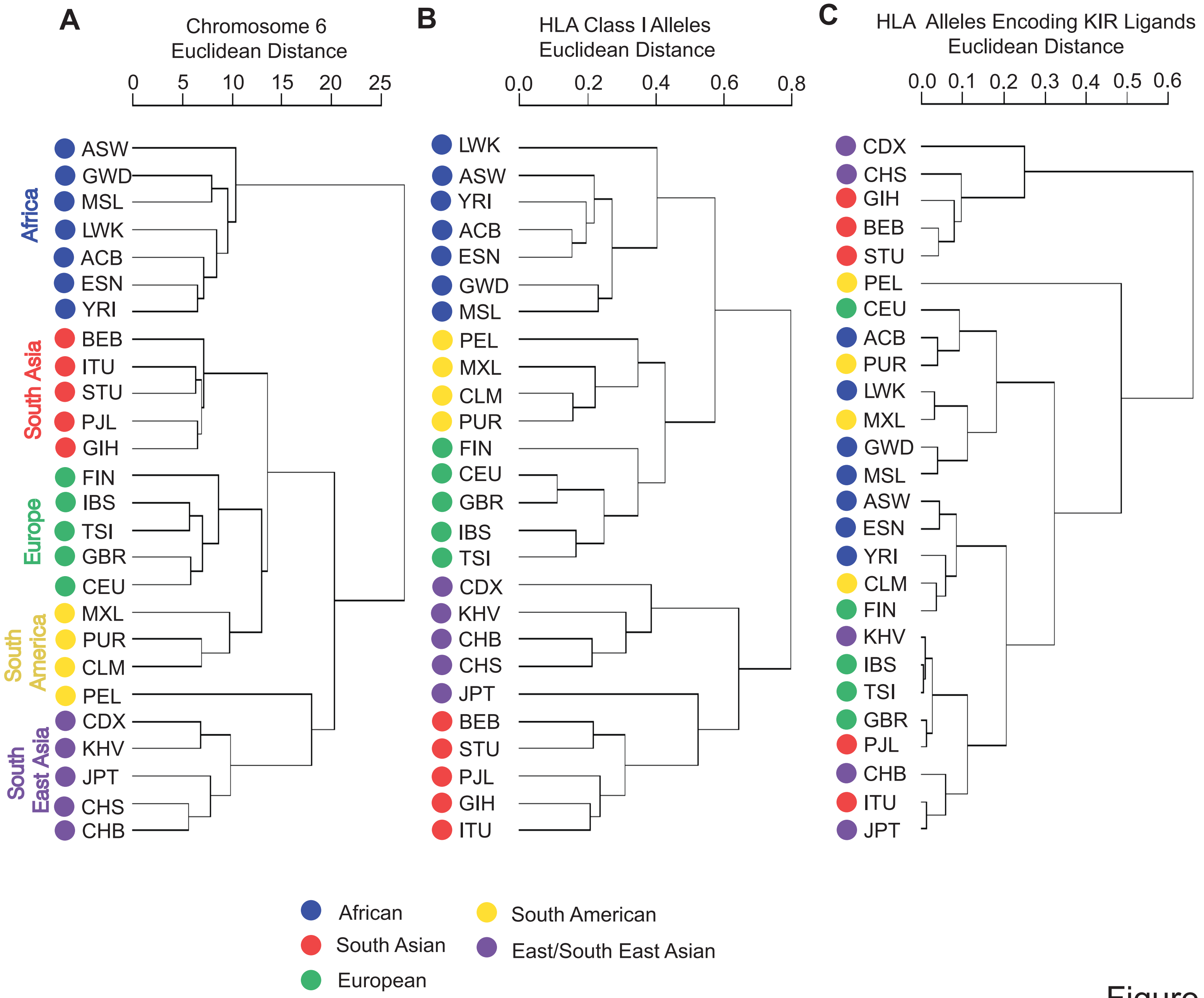

A
Chromosome 6
Euclidean Distance
0 5 10 15 20 25
ASW
GWD
MSL
LWK
ACB
ESN
YRI
BEB
ITU
STU
PJL
GIH
FIN
IBS
TSI
GBR
CEU
MXL
PUR
CLM
PEL
CDX
KHV
JPT
CHS
CHB
Africa
South Asia
Europe
South America
South East Asia
B
HLA Class I Alleles
Euclidean Distance
0.0 0.2 0.4 0.6 0.8
LWK
ASW
YRI
ACB
ESN
GWD
MSL
PEL
MXL
CLM
PUR
FIN
CEU
GBR
IBS
TSI
CDX
KHV
CHB
CHS
JPT
BEB
STU
PJL
GIH
ITU
C
HLA Alleles Encoding KIR Ligands
Euclidean Distance
0.0 0.1 0.2 0.3 0.4 0.5 0.6
CDX
CHS
GIH
BEB
STU
PEL
CEU
ACB
PUR
LWK
MXL
GWD
MSL
ASW
ESN
YRI
CLM
FIN
KHV
IBS
TSI
GBR
PJL
CHB
ITU
JPT
African
South Asian
European
South American
East/South East Asian

Figure S5

Centromeric *KIR*

3DL3	Freq.
*010	0.448
*008	0.180
*009	0.155
*006	0.046
*001	0.041
*015	0.033
† *04802	0.020
*028	0.018
† *02602	0.011
*048	0.011
*004	0.008
*002	0.005
*003	0.005
† *062	0.005
† *064	0.005
† *063	0.003
† *065	0.003
† *01003	0.002
*013	0.002

2DS2	Freq.
*00101	0.111
† *009	0.005
neg	0.884

2DL5B	Freq.
B*006	0.042
B*010	0.023
B*002	0.016
B*008	0.002
neg	0.917

2DS3/5	Freq.
5*00201	0.044
3*001	0.033
3*00201	0.008
neg	0.915

2DL2/3	Freq.
3*00101	0.701
3*00201	0.129
2*00301	0.100
3*023	0.021
2*00101	0.018
† 3*00109	0.007
3*015	0.003
3*022	0.003
† 3*028	0.003
† 3*00110	0.002
3*019	0.002
3*021	0.002
† 3*025	0.002
† 3*026	0.002
† 3*027	0.002
† 3*029	0.002
† 3*031	0.002
† 2*013	0.002

2DL1	Freq.
*00302	0.735
*00201	0.134
*00401	0.036
*001	0.002
† *00304	0.002
† *00305	0.002
† *030	0.002
† *031	0.002
† *033	0.002
† *034	0.002
neg	0.085

Telomeric *KIR*

2DL4	Freq.
*00102	0.498
*00501	0.163
*011	0.129
*006	0.082
*00801	0.052
*00103	0.044
† *00503	0.005
† *00504	0.003
† *032	0.002
† *033	0.002
† *034	0.002
neg	0.018

3DL1	Freq.
*01502	0.485
S1*01301	0.158
*00501	0.129
*00701	0.083
*00101	0.049
*020	0.041
*02901	0.016
† *070	0.003
† S1*082	0.003
† S1*085	0.003
*00502	0.002
*008	0.002
† *01505	0.002
† *079	0.002
† S1*078	0.002
† S1*083	0.002
† S1*084	0.002
neg	0.016

2DL5A	Freq.
A*001	0.114
A*005	0.052
A*012	0.007
† A*022	0.002
neg	0.825

2DS3/5	Freq.
5*00201	0.123
3*00201	0.049
neg	0.828

2DS4	Freq.
*00101	0.542
*010	0.127
*00401	0.083
*00301	0.052
† *018	0.003
† *00105	0.002
† *017	0.002
neg	0.188

2DS1	Freq.
*00201	0.175
*00202	0.003
*006	0.002
neg	0.820

3DL2	Freq.
*002	0.528
*010	0.119
*00701	0.108
*008	0.067
*001	0.057
*009	0.036
*039	0.031
*015	0.010
*021	0.010
† *093	0.010
*016	0.005
† *091	0.005
† *00706	0.003
† *00707	0.003
*027	0.003
† *083	0.002
† *084	0.002
† *099	0.002

Figure S6

Novel allele name	GenBank Accession	Most similar allele	Nucleotide change (position in full CDS)	Exon	Substitution (Codon Number)	Amino acid substitution	ref
*2DL1*00304*	KT438851	*2DL1*00302*	582 G > A	5	173 GGG > GGA	syn	
*2DL1*00305*	KT438852	*2DL1*00302*	1044 A > G	9	327 CCA > CCG	syn	
*2DL1*030*	KP025959	*2DL1*00302*	343 A > G	4	94 AGT > GGT	Ser > Gly	a
*2DL1*031*	KP025960	*2DL1*00302*	188 A > G	4	42 GAG > GGG	Glu > Gly	b
*2DL1*033*	KT438853	*2DL1*00302*	867 C > G	8	268 AGC > AGG	Ser > Arg	c
*2DL1*034*	KT438854	*2DL1*00302*	13 G > T	1	17 GTC > TTC	Val > Phe	d
*2DL2*013*	KM017076	*2DL2*00302*	806 C > G<br>1018 T > C	7<br>9	248 TCC > TGC<br>319 TCC > CCC	Ser > Cys<br>Ser > Pro	e
*2DL3*00109*	KF766495	*2DL3*00101*	478 C > T	5	139 CTA > TTA	syn	e
*2DL3*00110*	KF766497	*2DL3*00101*	618 A > C	5	185 CCA > CCC	syn	f
*2DL3*025*	KF766496	*2DL3*00101*	280 C > A	4	73 CTT > ATT	Leu > Ile	e
*2DL3*026*	KF766498	*2DL3*00101*	202 G > A	4	47 GAC > AAC	Asp > Asn	e
*2DL3*027*	KF766499	*2DL3*00101*	809 G > C	7	249 TGC > TCC	Cys > Ser	e
*2DL3*028*	KF766500	*2DL3*00101*	800 G > A	7	246 CGC > CAC	Arg > His	e
*2DL3*029*	KF766501	*2DL3*00101*	505 C > A	5	148 CGT > AGT	Arg > Ser	e
*2DL3*031*	KF849247	*2DL3*00101*	735 T > C<br>736 G > A	7<br>7	224 CAT > CAC<br>225 GTT > ATT	syn<br>Val > Ile	e
*2DL4*00503*	KT438855	*2DL4*00501*	888 G > A	9	273 CAG > CAA	syn	g
*2DL4*00504*	KT438856	*2DL4*00501*	987 G > T	9	306 GTG > GTT	syn	g
*2DL4*032*	KT438858	*2DL4*00102*	223 A > G	3	52 AAC > GAC	Asn > Asp	g
*2DL4*033*	KT438859	*2DL4*00102*	1012 C > T	9	315 CCC > TCC	Pro > Ser	g
*2DL4*034*	KT438857	*2DL4*00501*	200 G > T	3	44 GGG > GTG	Gly > Val	g
*2DL5A*022*	KT438863	*2DL5A*00501*	289 G > A	3	76 GGT > AGT	Gly > Ser	
*2DS2*009*	KT438862	*2DS2*00101*	109 C > T	4	16 CCC > TCC	Pro > Ser	
*2DS4*00105*	KP025962	*2DS4*00101*	657 C > T	5	198 TCC > TCT	syn	h
*2DS4*017*	KP025961	*2DS4*00101*	707 C > G	6	215 TCC > TGC	Ser > Cys	h
*2DS4*018*	KP025963	*2DS4*010*	316 C > G	4	85 CAC > GAC	His > Asp	h
*3DL1*01505*	KF849249	*3DL1*01502*	906 C > T	5	281 TAC > TAT	syn	i
*3DL1*079*	KF849250	*3DL1*01502*	1119 G > T	8	352 ATG > ATT	Met > Ile	i
*3DL2*00706*	KT899864	*3DL2*00701*	495 A > G	4	144 TCA > TCG	syn	j
*3DL2*00707*	KT899868	*3DL2*00701*	783 C > A	5	240 GCC > GCA	syn	j
*3DL2*083*	KT899867	*3DL2*008*	292 T > A	3	77 TCA > ACA	Ser > Thr	
*3DL2*084*	KT438861	*3DL2*00902*	502 G > A	4	147 GTT > ATT	Val > Ile	j
*3DL2*091*	KT438860	*3DL2*01001*	1315 A > C	9	418 AAA > CAA	Lys > Gln	j
*3DL2*093*	KT899866	*3DL2*00701*	532 G > A	4	157 GCC > ACC	Ala > Thr	
*3DL2*099*	KT899865	*3DL2*00902*	296 G > A<br>308 T > C	3<br>3	78 CGC > CAC<br>82 CTC > CCC	Arg > His<br>Leu > Pro	
*3DL3*01003*	KU529275	*3DL3*01001*	408 G > A	4	115 TCG > TCA	syn	
*3DL3*02602*	KU529271	*3DL3*026*	1074 A > G	8	337 CAA > CAG	syn	
*3DL3*04802*	KU529269	*3DL3*048*	1074 A > G	8	337 CAA > CAG	syn	k
*3DL3*062*	KU529272	*3DL3*026*	1074 A > G<br>1184 C > T	8<br>9	337 CAA > CAG<br>374 ACT > ATT	syn<br>Thr > Ile	
*3DL3*063*	KU529270	*3DL3*048*	1074 A > G<br>1184 C > T	8<br>9	337 CAA > CAG<br>374 ACT > ATT	syn<br>Thr > Ile	
*3DL3*064*	KU529273	*3DL3*00601*	1184 C > T	9	374 ACT > ATT	Thr > Ile	
*3DL3*065*	KU529274	*3DL3*00102*	1184 C > T	9	374 ACT > ATT	Thr > Ile	
*3DS1*078*	KJ001806	*3DS1*01301*	775 G > C	5	238 GGG > CGG	Gly > Arg	i
*3DS1*082*	KJ001804	*3DS1*01301*	1114 T > C	8	351 GCT > GCC	syn	i
*3DS1*083*	KJ001805	*3DS1*01301*	393 T > G<br>400 G > C<br>416 G > C	4<br>4<br>4	110 GGT > GGG<br>113 GTG > CTG<br>118 AGA > ACA	syn<br>Val > Leu<br>Arg > Thr	i
*3DS1*084*	KJ001807	*3DS1*01301*	308 C > T	3	82 CCC > CTC	Pro > Leu	i
*3DS1*085*	KJ365317	*3DS1*01301*	934 C > T	5	291 CTT > TTT	Leu > Phe	i

Figure S7
